# An *in silico* hiPSC-Derived Cardiomyocyte Model Built With Genetic Algorithm

**DOI:** 10.3389/fphys.2021.675867

**Published:** 2021-06-16

**Authors:** Akwasi D. Akwaboah, Bright Tsevi, Pascal Yamlome, Jacqueline A. Treat, Maila Brucal-Hallare, Jonathan M. Cordeiro, Makarand Deo

**Affiliations:** ^1^Department of Engineering, Norfolk State University, Norfolk, VA, United States; ^2^Masonic Medical Research Institute, Utica, NY, United States; ^3^Department of Mathematics, Norfolk State University, Norfolk, VA, United States

**Keywords:** biophysical model, genetic algorithm, hiPSC-derived cardiomyocytes, computational biology, cardiac electrophysiology

## Abstract

The formulation of *in silico* biophysical models generally requires optimization strategies for reproducing experimentally observed phenomena. In electrophysiological modeling, robust nonlinear regressive methods are often crucial for guaranteeing high fidelity models. Human induced pluripotent stem cell-derived cardiomyocytes (hiPSC-CMs), though nascent, have proven to be useful in cardiac safety pharmacology, regenerative medicine, and in the implementation of patient-specific test benches for investigating inherited cardiac disorders. This study demonstrates the potency of heuristic techniques at formulating biophysical models, with emphasis on a hiPSC-CM model using a novel genetic algorithm (GA) recipe we proposed. The proposed GA protocol was used to develop a hiPSC-CM biophysical computer model by fitting mathematical formulations to experimental data for five ionic currents recorded in hiPSC-CMs. The maximum conductances of the remaining ionic channels were scaled based on recommendations from literature to accurately reproduce the experimentally observed hiPSC-CM action potential (AP) metrics. Near-optimal parameter fitting was achieved for the GA-fitted ionic currents. The resulting model recapitulated experimental AP parameters such as AP durations (APD_50_, APD_75_, and APD_90_), maximum diastolic potential, and frequency of automaticity. The outcome of this work has implications for validating the biophysics of hiPSC-CMs in their use as viable substitutes for human cardiomyocytes, particularly in cardiac safety pharmacology and in the study of inherited cardiac disorders. This study presents a novel GA protocol useful for formulating robust numerical biophysical models. The proposed protocol is used to develop a hiPSC-CM model with implications for cardiac safety pharmacology.

## Introduction

High fidelity numerical biophysical models have the potential to provide the missing mechanistic link between the experimental observations and their clinical implications. Formulating such models are primarily optimization problems with the experimental data as targets. Metaheuristic algorithms, such as genetic algorithms (GAs; Smith et al., 1995), are well suited for this task due to their inherent stochastic and judicious exploration of the solution space. In electrophysiological studies, single- and multi-cell mathematical models have aided efforts at elucidating various biological processes, such as perception, cognition, and cardiac function, which is the focus of this study.

Human induced pluripotent stem cells (hiPSCs), since their discovery by Takahashi et al. (2007), have been instrumental at the development of ethically sound patient-specific human cell and tissue models. These models have, in turn, allowed scientists to investigate the underpinnings of congenital and drug-induced disorders. A prominent derivative of this cell type is the human induced pluripotent stem cell-derived cardiomyocyte (hiPSC-CM). hiPSC-CMs have recently proven to be both useful and promising in cardiac safety pharmacology, where these cells are adopted as test benches for studying drug effects on cardiac function (Gintant et al., 2019). hiPSC-CMs have also been used to better understand drug-induced and inherited cardiac disorders such as long QT syndrome (Moretti et al., 2010; Egashira et al., 2012) and catecholaminergic polymorphic ventricular tachycardia (Fatima et al., 2011; Jung et al., 2012). Prior to the advent of hiPSC-CMs, researchers often relied on animal models to make extrapolations of disease effects in human cells. This route possesses a significant caveat of dissimilar genotypic representation by these models. While freshly excised human cardiac cells are the ideal candidates for inductive human cardiac studies, obtaining these uncommon cells is highly invasive relative to hiPSC-CMs, which can even be derived from superficial somatic cells such as skin cells. Although hiPSC-CM is apparently a convenient choice for extensive long- and short-term cardiac studies, there are some deficiencies in the electrophysiological properties. There are ongoing attempts by scientists to better understand these cells and validate them as viable substitutes for human cardiomyocytes. These attempts are mainly *in vitro* and *in silico*. For instance, the Comprehensive *in vitro* Proarrhythmia Assay initiative, first presented in 2013, stipulates research directions around the use of hiPSC-CM experimental data and mathematical modeling in proarrhythmic risk assessment (Gintant et al., 2017).

Since the pioneering work by Hodgkin and Huxley (1952) involving the formulation of a biophysical mathematical model of the squid giant axon, there have been numerous attempts at creating biophysical models for different cell types (Di Francesco and Noble, 1985; Luo and Rudy, 1994; Courtemanche et al., 1998; Kurata et al., 2002). These models have been helpful at elucidating the electrical dynamics of native cardiomyocytes. Recently, there have been few attempts at formulating *in silico* hiPSC-CM models (Paci et al., 2013, 2018; Koivumäki et al., 2018; Kernik et al., 2019). However, the inherent variability in these cells pose challenges to deriving formulations that could reproduce the wide range of behaviors of hiPSC-CMs. One recent approach is to combine experimental data from multiple labs to derive model formulations on averaged data (Kernik et al., 2019). However, care must be taken while combining data from disparate sources because the inconsistencies in cell origins (Hwang et al., 2015), culturing environments, experimental protocols and conditions, as well as cell maturation levels (Narsinh et al., 2011) may introduce unwanted deviations in the model. This study joins in the efforts at formulating a robust *in silico* hiPSC-CM model based mostly on the experimental data from a single lab for maintaining phenotypical consistency. In achieving this, we present a novel customizable GA protocol employed for a near-optimal fitting of model ionic current formulations to the experimental data.

Genetic algorithm is a heuristic optimization method inspired by Darwinian evolution and natural selection (Smith et al., 1995). The process, similar to the biological counterpart, involves population (i.e., sets of possible solutions termed chromosomes) initialization to crossover, which involves a combinatorial shuffling of parameters (termed genes) about a specified number of pivot points (termed crossover points) between two parent solutions to generate offspring. Offspring solutions may then undergo mutation, typically done in an additive or multiplicative fashion. A fitness criterion must be defined to facilitate the propagation of near-optimal solutions over the specified number of iterations termed generations. Metaheuristic optimization methods offer resilience against local optima and saddle point convergence relative to gradient-based nonlinear optimization methods, such as the Newton–Raphson and Levenberg–Marquardt methods (Rhinehart, 2016). GA-based parametrization has been used to fine-tune mathematical models of murine myocytes (Bot et al., 2012; Groenendaal et al., 2015) and canine atrial cells (Syed et al., 2005) to incorporate cell-specific experimental data.

Our previous work (Akwaboah et al., 2020) demonstrated the feasibility of using GA to fit cardiac cell biophysical model formulations. In this study, we demonstrate how the GA-based ionic current fitting outcomes can be further integrated into the biophysical numerical model development process. An improved GA-based parametrization protocol was developed to build a complete *in silico* biophysical model of a hiPSC-CM.

## Materials and Methods

### Preparations of hiPSC-CMs

Human induced pluripotent stem cells usage was approved by the Institutional Stem Cell Research Oversight committee at Masonic Medical Research Institute. hiPSCs (from WiCell) were maintained on growth factor-reduced Matrigel coated plates in E8 medium with E8 supplement. Cardiac differentiation was induced by 6 μM CHIR99021, 10 ng/ml Activin A, and 5 nM rapamycin in RPMI1640 medium containing B27 (minus insulin) and 50 μg/ml L-ascorbic acid (cell culture tested powder) as the basal medium. After 24 h, media was changed in the same basal medium. The following day, cells were kept in RPMI1640/B27 (-insulin) with the addition of 5 μM XAV939 and 10 μM KY0211 for days 2–6, changing the media every other day. Afterward, cells were kept in RPMI1640/B27 (+insulin) with 50 μg/mL L-ascorbic acid for days 8–10, followed by a purification medium, RPMI 1640 without glucose, and supplemented with 4 mM sodium L-lactate. Cells were kept in the purification medium for days 12–14, then back to the basal medium of RPMI/B27(+insulin) supplemented with 20 ng/ml triiodothyronine (T3) and 1 μM dexamethasone until day 30. For single myocytes, the monolayers were then dissociated around day 25 with 0.05% trypsin, 1 mg/ml collagenase II, and plated onto Matrigel coated dishes. All voltage clamp recordings were made 5 days after recovery. Experiments were typically performed on hiPSC-CMs at least 20 days post-differentiation.

### Voltage Clamp Recordings

**FIGURE 1:**
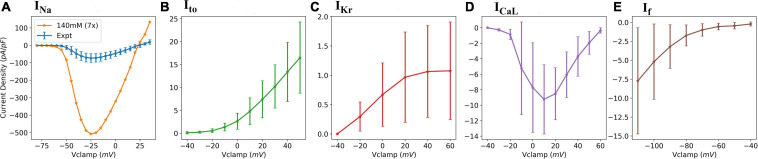
Experimental data IV plots. **(A)** fast sodium current (*I*_Na_); **(B)** transient outward potassium current (*I*_to_); **(C)** delayed rectifier potassium current (*I*_Kr_); **(D)** L-type calcium current (*I*_CaL_); and **(E)** Hyperpolarization-activated current (*I*_f_). The errors bars represent the experimental standard deviations from multiple cell recordings.

Whole-cell patch clamp recordings were obtained for five ionic currents, namely: fast sodium current, *I*_Na_; transient outward potassium current, *I*_to_; L-type calcium current, *I*_CaL_; rapid delayed rectifier potassium current, *I*_Kr_; and hyperpolarization-activated current, *I*_f_. Figure 1 presents the experimental current-voltage (IV) plots for all five currents.

*I*_Na_ was measured as described previously (Goodrow et al., 2018). Briefly, the bath solution consisted of 2 mM CaCl_2_, 10 mM glucose, 1 mM MgCl_2_, 105 mM N-Methyl D Glucamine, 40 mM NaCl, and 10 mM HEPES free acid. The pH was adjusted to 7.4 with HCl. Also, a 300 μM CdCl_2_ is added to block the calcium currents which may interfere with *I*_Na_ recording. The pipette solution was composed of 1 mM MgCl_2_, 15 mM NaCl, 5 mM KCl, 120 mM CsF, 10 mM HEPES, and 10 mM EGTA. Before the experiments, 5 mM Na_2_ATP was added, and the pH was adjusted to 7.2 by the addition of CsOH. *I*_Na_ was recorded by applying command voltages (25 ms-long pulses) in steps of 5 mV over the range of –80 – +35 mV from a holding potential of –120 mV. All *I*_Na_ measurements (*n* = 8–15) were taken at 20°C and a lowered extracellular (bath) sodium concentration of 40 mM to ensure an adequate voltage control. Temperature and concentration extrapolation facilitated by the Q_10_ (temperature adjustment factor) correction and Goldman–Katz constant field equation was predicted by Goodrow et al. (2018) to be a factor of 7 and was subsequently adopted in the *I*_Na_ curve fitting. More details about the *I*_Na_ experimental protocol can be found in Goodrow et al. (2018). *I*_Kr_ was recorded as described previously in Treat et al. (2019). In recording the potassium currents, the conventional K^+^ pipette solution of 90 mM K^+^-aspartate, 45 mM KCl, 10 mM NaCl, 1 mM MgCl_2_, 10 mM HEPES, 5 mM EGTA, and 5 mM MgATP, with a pH of 7.2 (maintained by adding KOH), was used. *I*_Kr_ was recorded by applying 300 ms-long test pulses between –40 and +60 mV in steps of 20 mV from a –80 mV holding potential as described in Doss et al. (2012). *I*_CaL_ was recorded using 300 ms-long test pulses applied between –40 and +60 mV in steps of 10 mV from a holding potential of –40 mV. *I*_to_ was measured as described in Cordeiro et al. (2013). Briefly, the voltage clamp protocol consisted of a holding potential of –80 mV, followed by a brief –50 mV potential to ensure that all sodium channels were inactivated. This is important as *I*_to_ closely follows the depolarization facilitated by Na^+^ influx. Test pulses were then applied in steps of 10 mV from –40 to +50 mV for each clamp voltage. The half-inactivation voltage of *I*_to_, *V*_1/2_ = −41.1 ± 0.2mV, and a slope, *k* = 6.68± 0.19, were used in our model based on previous studies (Cordeiro et al., 2013). *I*_f_ was recorded using a holding potential of –40 mV, followed by pulses from –110 to –40 mV in steps of 10 mV.

All voltage clamp recordings were made using a MultiClamp 700A (Molecular Devices, Sunnyvale, CA, United States). Whole cell patch pipettes were made from glass capillary tubes (1.5 mm O.D., Fisher Scientific, Pittsburg, PA, United States) and pulled on a gravity puller (Narishige Corporation, East Meadow, NY, United States). The resistance ranged from 1.0 to 3.0 MΩ and electronic compensation of series resistance was applied (∼60–70%). Capacitance of the hiPSC-CMs was measured by applying –5 mV voltage steps. Signals were acquired at sampling rate of 10–25 kHz, filtered at 4–6 kHz, and digitized with a Digidata 1322 converter (Molecular Devices, Sunnyvale, CA, United States). Drugs were rapidly applied to the cell using a four-barrel quartz micromanifold (ALA Scientific Instruments Inc., Westbury, NY, United States) placed 500 μm from the cell. Acquisition and analysis were performed using the pClamp9 software. All hiPSC-CM experiments were performed at 36°C, except peak *I*_Na_ which was recorded at room temperature (20°C).

Pooled data are presented as Mean ± SEM. Statistical analysis was performed using an ANOVA followed by a Student–Newman–Keuls test using the SigmaStat software.

### Mathematical Model of hiPSC-CM

**FIGURE 2:**
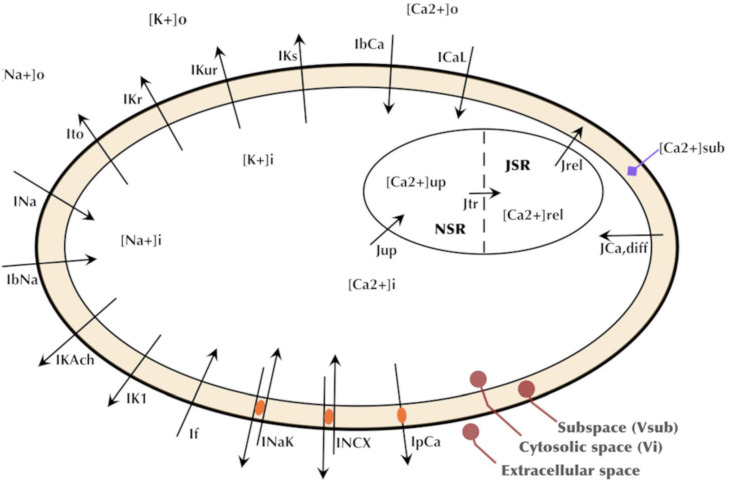
Schematic of the hiPSC-CM model depicting the major ionic currents and fluxes.

Formulations from six existing cardiac cell models were adopted based on being representative of human cardiomyocyte electrophysiology and/or reproducing spontaneous activity. The formulations of five key currents (*I*_Na_, *I*_to_, *I*_CaL_, *I*_Kr_, and *I*_f_) were optimized by GA based on the experimental data acquired in our lab. The rest of the current formulations, namely, ultrarapid, and slow delayed potassium rectifier currents (*I*_Kur_ and *I*_Ks_), sodium-calcium exchanger current (*I*_*NCX*_ or *I*_*NaCa*_), sodium-potassium exchange pump current (*I*_*NaK*_), calcium pump current (*I*_*pCa*_), background sodium and calcium currents (*I*_*bNa*_ and *I*_*bCa*_), acetylcholine-activated inward-rectifying potassium current (*I*_KAch_), and inward rectifier current (*I*_*K1*_), were adjusted through proportional scaling based on published literature. The patch clamp experiments in our and other laboratories have revealed a very low to negligible *I*_*K1*_ in hiPSC-CMs (Doss et al., 2012; Bett et al., 2013; Vaidyanathan et al., 2016). Similarly, most of the hiPSC-CMs in our lab do not express *I*_Ks_. The experimental study by Ma et al. (2011) also reported *I*_Ks_ in only five out of 16 cells, and its role in AP repolarization was insignificant. Therefore, we did not include *I*_*K1*_ and *I*_Ks_ in the GA-based optimization. The *I*_Na_ formulation was adopted from the Luo–Rudy model (Luo and Rudy, 1994), which is a widely adopted mammalian ventricular myocyte model formulated based on the Hodgkin–Huxley formalism. The *I*_to_ formulation was adopted from the Grandi–Pandit human atrial cell model (Grandi et al., 2011) and modified based on the experimental data acquired in hiPSC-CMs by Cordeiro et al. (2013). The *I*_f_ formulation was adopted from the human cardiac Purkinje cell model by Stewart et al. (2009). *I*_CaL_ and *I*_Kr_ were formulated based on the mammalian sinoatrial nodal cell model by Kurata et al. (2002). The choice of this model is due to the inherent automaticity exhibited by hiPSC-CMs similar to that of the nodal cells. The intracellular calcium dynamics in hiPSC-CMs is similar to the nodal cells due to the lack of a mature T-tubules structure (Li et al., 2013). Therefore, the intracellular calcium handing [involving the Ca^2+^ release and uptake fluxes between the sarcoplasmic reticulum (SR) and the cytosol as well as the intra-SR Ca^2+^ transfer flux occurring between the junctional SR and network SR] was adopted from the Kurata model as well. Figure 2 presents a schematic of our hiPSC-CM model depicting the constituent ionic currents and fluxes. Table 1 lists the references to the adopted ionic current formulations. The transmembrane voltage, *V*_*m*_, is given by the following first order differential equation:

**TABLE 1:** Summary of sources (References) to adopted model formulations.

Ionic current(s)	Formulation source
*I*_Kur_, *I*_*NaCa*_, *I*_*NaK*_, *I*_*pCa*_, *I*_*bNa*_, *I*_*bCa*_, *I*_Ks_	Courtemanche et al., 1998
*I*_Na_	Luo and Rudy, 1994
*I*_Kr_, *I*_KACh_	Kurata et al., 2002
*I*_CaL_	Kurata et al., 2002; ten Tusscher et al., 2004
*I*_to_, *I*_*K1*_	Grandi et al., 2010
*I*_f_	Stewart et al., 2009

(1) $$C_{m}\frac{dV_{m}}{dt}=-\sum_{i}I_{{\text{ion}}_{i}}$$

where, *C*_*m*_ is the membrane capacitance and *I*_*ion_i*_ is an instance of the 14 ionic current formulations constituting the model in this study.

### Numerical Implementations: Ionic Current Formulations and Whole Cell Integration

In the *in silico* implementation of current formulations, computational equivalents of the experimental voltage clamping protocols for the five ionic currents mentioned earlier were implemented. An initial forward Euler implementation was done for all five currents; various time steps were tried, where a sufficiently small time-step for computational stability meant a prolonged runtime, as in the case of the rapid *I*_Na_ and *I*_CaL_ currents, we deferred to an adaptive solver. The *I*_Na_ model was simulated using the implicit backward difference (BDF) integration (Iserles, 2008) by implementing voltage test pulses from –80 to +35 mV in the steps of 5 mV with a duration of 25 ms (similar to the experimental protocol). The peak inward current values were then identified and used to produce IV plots. The BDF employed an adaptive computational time-step; where signal gradients were high (as in the case of the rapid depolarization), a suitably small time-step is automatically adopted and *vice versa*. This allowed for an accelerated computational runtime with a minimal approximation error in single run and multi-generational GA fitting processes. The *I*_Kr_ voltage clamp simulations relied on a forward Euler integration with a computational time-step of 0.2 ms. The chosen time values ensured a trade-off between high resolution time discretization and extended time spans. Voltage pulse intensities applied from a –80-mV holding potential ranged from –40 to +60 mV in steps of 20 mV at a 300 ms pulse duration. Peak *I*_Kr_ tail currents were measured at –50 mV. Voltage clamping for *I*_CaL_ was simulated using the implicit, adaptive time step, BDF integration. The characteristic equations were modified based on ten Tusscher et al. (2004) to allow the dependence on a dynamic Ca^2+^ reversal potential, rather than the fixed-Ca^2+^-potential formalism adopted in the original Kurata model (Kurata et al., 2002). Application of 500 ms voltage pulses ranging from –40 to +60 mV were applied from a holding potential of –30 mV, similar to the experimental protocol. *I*_to_ voltage clamp simulations were performed by the explicit forward Euler integration with a fixed time step of 0.5 ms. Voltage pulse intensities applied from a holding potential of –80 mV followed a brief –50 mV potential (as described in the experimental protocol) ranged from –40 to +50 mV in steps of 10 mV. Pulse duration of 500 ms was adopted. *I*_f_ implementation was executed by the forward Euler integration with a fixed time step of 10 ms. Voltage pulses that are 500 ms long were applied from a holding potential of –40 mV over a range of –110 to –40 mV in 10 mV increments.

We performed whole cell simulations using the implicit Radau adaptive integration method provided by the solve_ivp module in the SciPy python package. Alternatively, we implemented a faster C/C++ version, where a forward Euler integration was employed.

To enable the pacing of the model by an external stimulus of varying frequencies, the spontaneous firing of APs was disabled by decreasing the maximum conductance of *I*_f_ by 50%. The effects of adrenergic stimulation using isoproterenol were simulated by altering the maximum conductance of five currents as described previously (Shah et al., 2019). Briefly, the conductance of L-type Ca^2+^ channel (*I*_CaL_), Na^+^/K^+^ pump (*I*_*NaK*_), Na^+^/Ca^2+^ exchanger (*I*_*NaCa*_), and SR Ca^2+^-ATPase (SERCA) were up-regulated by 100, 30, 30, and 20%, respectively, while *I*_*K1*_ was down-regulated by 20%. The effects of cholinergic stimulation using acetylcholine (ACh or CCh) were simulated by scaling the maximum conductance of *I*_KACh_ by 200%. The effects of 4-aminopyridine (4-AP) were simulated by varying levels of *I*_Kur_ block.

### Model Parametrization

Characteristic equations for the five currents were parametrized, over which, the optimization was done. For each ionic current optimization, parametrization was executed by converting scaling coefficients (*a*_*xi*_) and half-activation/inactivation voltages (*b*_*xi*_) and slopes (*c*_*xi*_) of the sigmoidal gating equations into free parameters as presented in Eq. (2) below.

(2) $$\alpha_{xi}\left(V_{m}\right)|\beta_{xi}\left(V_{m}\right)=\frac{a_{\mathrm{xi}}\left(V_{m}-b_{\mathrm{xi}}\right)}{\text{exp}\left(\frac{V_{m}-b_{\mathrm{xi}}}{c_{\mathrm{xi}}}\right)}$$

Parameter sets for each current were bundled as chromosomes and optimized heuristically by the GA (described next). Accordingly, 20, 18, 20, 6, and 7 free parameters were created for the equations of *I*_Na_, *I*_to_, *I*_Kr_, *I*_CaL_, and *I*_f_, respectively. Detailed parametric equations for each current are given in the Supplementary Material.

### The Genetic Algorithm Protocol

Myriads of GA protocols can be formulated by adjusting the population size, fitness criterion, number of generations, crossover, and mutation schemes. A description of the GA protocol adopted and its implementation are discussed in this section.

A starting population was initialized by generating individuals composed of genes selected randomly from a uniformly distributed interval. Constraints (interval limits) were chosen such that the existing model parameter values were contained in the range. The rationale behind this was to initialize the search from a physiologically feasible solution space, as there exists the caveat of multiple individual solutions producing the same/similar model effects. The initial population is, thus, governed mathematically by:

(3) $$\rho^{\left\{0\right\}}\in \left[\left(1-\lambda_{a}\right)\theta ,\left(1+\lambda_{a}\right)\theta \right]$$

where, ρ^{0}^ is the stochastic-drawn initial population parameter set, λ_*a*_ is the initial population gene range width determination constant, and θ is the original model parameter. The condition was applied to genes in all chromosomes in the generation. Superscripts in format {*x*} throughout this paper indicates the generation number.

**FIGURE 3:**
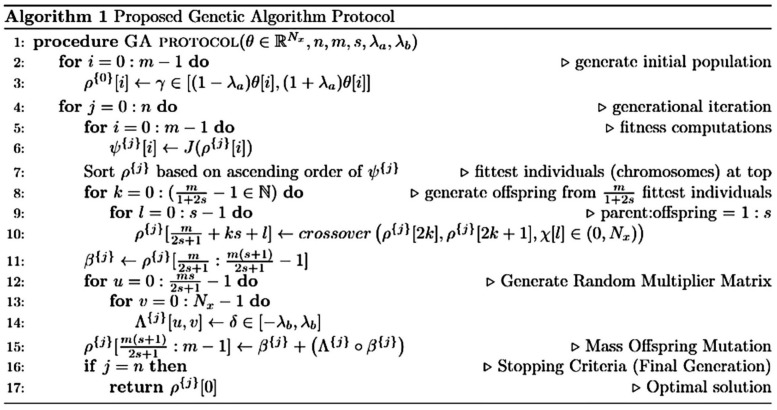
Algorithmic description of the genetic algorithm (GA) protocol used in fitting the ionic currents. θ: original model chromosome; θ[*i*]: original model gene; *N*_*x*_: chromosomal length/number of genes; *m*: population size; *s*: offspring proportion (parent-offspring ratio =  1:*s*); *J*(): loss function (SSE or RMSE); λ_*a*_: initial population constraint; λ_*b*_: mutation constraint; and χ: an iterable comprising lists of crossover point(s). Length(χ[*i*]) ∈ (0,*s*]. All intervals [i.e., [a, b], (a, b] or (a, b)] are uniformly distributed. Parameters belonging to these intervals were randomly drawn.

Population size of 10 × *Chromosome size* was chosen based on the recommendation by Storn (1996). However, an exception of reduced population size was made in the case of an extended computational runtime, which, in turn, was limited by the number of processors available [28 cores per node for the Ohio Supercomputer HPC clusters (Ohio Supercomputer Center (OSC), 1987) and 24 cores per node for the Extreme Science and Engineering Discovery Environment HPC (Towns et al., 2014)]. A detailed algorithmic description of the GA protocol used in fitting the ionic currents is presented in Figure 3.

The SSE loss or RMSE loss for the IV plot points between the model output and experimental data was adopted as the objective function to be minimized to ensure a robust curve fitting. For adaptive time-step, ordinary differential equation solvers available in the SciPy solve_ivp python module—implicit BDF and Radau (Guglielmi and Hairer, 2001) methods as well as explicit Runge–Kutta methods (Dormand and Prince, 1980; Petzold, 1983)—were used. The ionic current models were implemented as importable modules to allow parallel computation using the python multiprocessing module.

Mathematically, the objective function for individual fitness computation, *J*(θ_*n*_,*X*) can be expressed as:

(4) $$J_{\text{SSE}}\left(\theta_{n},X\right)=\sum_{i=0}^{T}{\left[X-I\left(\theta_{n}\right)\right]}^{2}$$

(5) $$J_{\text{RMSE}}\left(\theta_{n},X\right)=\sqrt{\frac{1}{T}\cdot \sum_{i=0}^{T}{\left[X-I\left(\theta_{n}\right)\right]}^{2}}$$

where, θ_*n*_ is symbolic of a parameter set, *X* is a set of experimental data points, *I*(θ_*n*_) represent the ionic current modeled as a function of the parameter set, and *T* is the number of data points (IV plots).

Single- and multi-point crossover scheme were adopted in fitting the five currents. For each offspring production, crossover points were stochastically drawn from a uniformly distributed interval of positive integers not exceeding the chromosomal length. The convention was to use a larger number of crossover points to compensate for a small population size relative to the number of parameters. Crossover involved the selection of multiple mating pairs from the pool of the best performing individuals. Genes in these mating pairs were then shuffled about the selected crossover-points. The convention chosen here was to generate the same number of offspring as the number of crossover points. This convention, though arbitrary, was sufficient at producing a reasonable fitting. It, however, does not indicate the maximum allowable number of unique offspring. The mutation protocol adopted in this work is an additive scheme involving addition of positive or diminutive proportions of the present offspring parameters to the offspring. The value of these proportions, like in the case of the initial population generation, are randomly selected from a uniformly distributed range. This can be expressed in a matrix form as:

(6) $$M=O+\left(\Lambda^{{}^{\circ}}O\right)$$

where, *M* is the resulting mutant matrix, *O* is the offspring matrix, and Λ is the proportion matrix. The constraints (mutation coefficient range width determination constant, *λ*_*b*_) for the elements in the proportion matrix is given mathematically as:

(7) $$\Lambda_{uv}^{\left(j\right)}\in \left[-\lambda_{b},\lambda_{b}\right]$$

The proportion-offspring multiplication in Eq. (6) is not a traditional matrix multiplication, but rather the Hadamard element-wise multiplication (*u* and *v* are row and column indices, respectively, whereas *j* is generation/iteration number). This way, multi-gene mutations involving all genes per chromosome are executed with random proportions simultaneously. After the two operations, element-wise multiplication and matrix addition, the mutant matrix picks on the offspring size, η = *sr*; where *r* is the parent population size. The parent-offspring-mutant ratio is therefore 1:*s*:*s* and, consequently, the population size, *m* = *r*(1 + 2*s*). A summary of the various GA protocols adopted in curve fitting for the five experimental-data-complemented ionic currents are presented in Table 2. In all fittings, the GA was run for 100 generations.

**TABLE 2:** Summary of the genetic algorithm protocols for ionic currents.

	*I*_Na_	*I*_to_	*I*_Kr_	*I*_CaL_	*I*_f_
# Parameters	20	20	18	6	7
Population size	27	80	25	54	80
Crossover	4-point	2-point	2-point	1-point	2-point
λ_*a*_	0.8	0.8	0.2	0.5	0.5
Mutation	All genes	All genes	All genes	All genes	All genes
λ_*b*_	0.2	0.5	0.1	0.1	0.2
Fitness	RMSE	SSE	SSE	SSE	SSE

To quantitatively ascertain the goodness of fit at the end of the GA, the coefficient of determination, *R*^2^, was used. This statistical measure gives the degree of variability in the data accounted for by the fitted model. The mathematical formula for *R*^2^ is given in Eq. (8). This value typically ranges between 0 and 1 (it may also be negative in the case of nonlinear regression), with 1 interpreted as an absolute fit accounting for 100% of the variability in the data captured by the fitted model:

(8) $$R^{2}=1-\frac{\sum^{N}{\left(y_{i}-\hat{y}\right)}^{2}}{\sum^{N}{\left(y_{i}-\bar{y}\right)}^{2}}=1-\frac{\text{SSE}}{\text{SST}}$$

where, *y*_*i*_ is experimental (actual) current values, $\hat{y}$ is the model fitted current value, and $\bar{y}$ is the mean of the experimental values. SSE and SST are the model residual sum of squared error and total sum of squared errors for the experimental data, respectively.

Once the five abovementioned currents were optimized by GA, the rest of the currents were manually scaled based on published literature to obtain the experimentally recorded AP morphology. The scaling used for the ionic currents and cell related constants are listed in Supplementary Tables 7, 8. Code implementation of the GA parameter optimization and the hiPSC-CM biophysical model are available at https://github.com/Adakwaboah/hiPSC-CM_Computational_Model.

## Results

### GA-Based Optimizations

**FIGURE 4:**
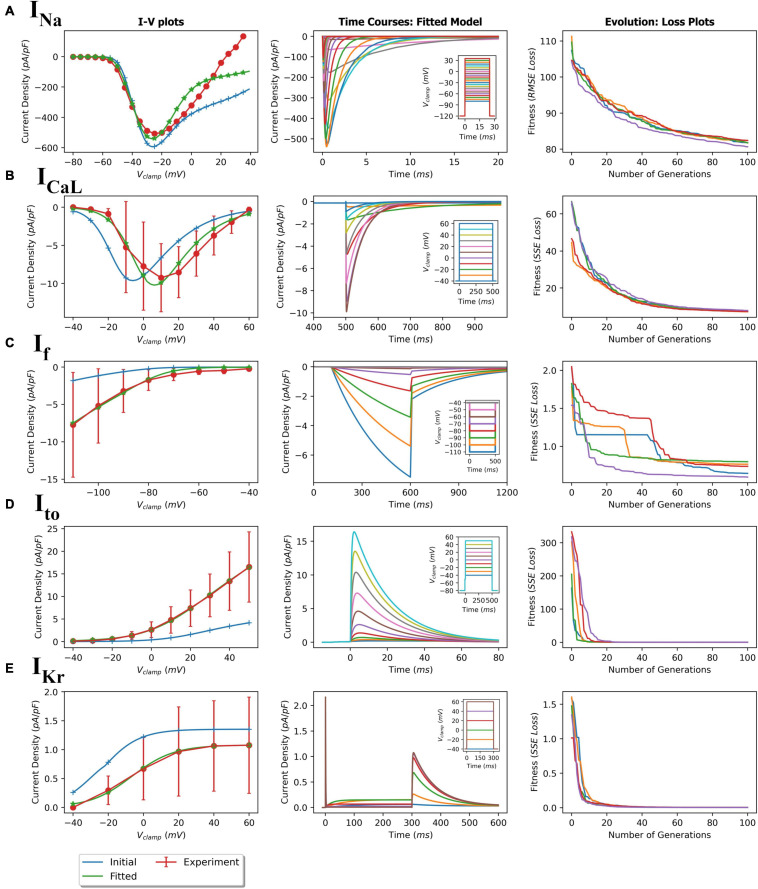
GA-based parameter fitting of ionic currents. **(A)** fast sodium current (*I*_Na_); **(B)** L-type calcium current (*I*_CaL_); **(C)** Hyperpolarization-activated current (*I*_f_); **(D)** transient outward potassium current (*I*_to_); and **(E)** delayed rectifier potassium current (*I*_Kr_). The left plots show initial and final fitted I-V plots for each current compared with the corresponding experimental values. Middle plots show the simulated time course of current activations based on the corresponding voltage clamp protocols (insets). Right plots show loss (RMSE or SSE) over 100 generations showing convergence during the GA fitting process.

Using the protocols described in the Methods section, parameters of the five ionic current formulations (*I*_Na_, *I*_to_, *I*_CaL_, *I*_Kr_, and *I*_f_) were optimized to reproduce the experimental data. The GA process for each ionic current being fitted were performed multiple times to ensure consistency and reproducibility of this meta-heuristic. For each of the fittings, the initial and final model IV plots (representative) are shown in Figure 4 (left panels). In addition, representative time course plots for each fitted current model obtained using simulated patch clamp protocols are shown in the middle panels. The fitted formulations were able to reproduce the experimental mean current recordings (see Supplementary Figure 7 for representative recordings). The right panels in Figure 4 show the near-optimal parameter convergence in the form of losses over 100 generations for the various GA trails per current. The apparent non-uniform decreasing trend in loss plots is evident of the stochastic yet perpetual search for the global minimum. Table 3 summarizes the initial and final fitted loss metrics (mean and standard deviations for multiple trials, *n* = 5) for all currents. Improvement in fitting can be seen in the increasing *R*^2^ values toward unity. The original and fitted parameter values for all currents along with the corresponding detailed formulations are given in the Supplementary Tables 1–6. Supplementary Figures 1–4, 6 compare the current activation/inactivation kinetics and corresponding time constants in our optimized model with two recent hiPSC-CM numerical models, namely, models from Paci et al. (2018) and Kernik et al. (2019). Computational runtimes for a single GA trial over the 100-generation period with parallel fitness computation over 28 cores were 11, 13, 7, 12, and 12 min for *I*_Na_, *I*_CaL_, *I*_f_, *I*_to_, and *I*_Kr_, respectively.

**TABLE 3:** Loss and fitness values of the GA-based optimization of currents.

Ionic current	Initial loss	Final loss	Initial *R*^2^ (initial model)	Final *R*^2^ (fitted model)
*I*_Na_	107.449 ± 2.76	81.661 ± 0.56	0.519	0.841 ± 2.18e-3
*I*_to_	288.313 ± 44.792	0.105 ± 0.038	–0.165	0.999 ± 1.19e-4
*I*_Kr_	1.403 ± 0.215	0.00467 ± 5.15e-4	–1.558	0.995 ± 5.16e-4
I_f_	0.708 ± 0.076	1.845 ± 0.186	–0.201	0.986 ± 1.49e-3
*I*_CaL_	57.892 ± 10.048	7.435 ± 0.224	0.358	0.9405 ± 1.8e-3
				

### Simulated Action Potentials

**FIGURE 5:**
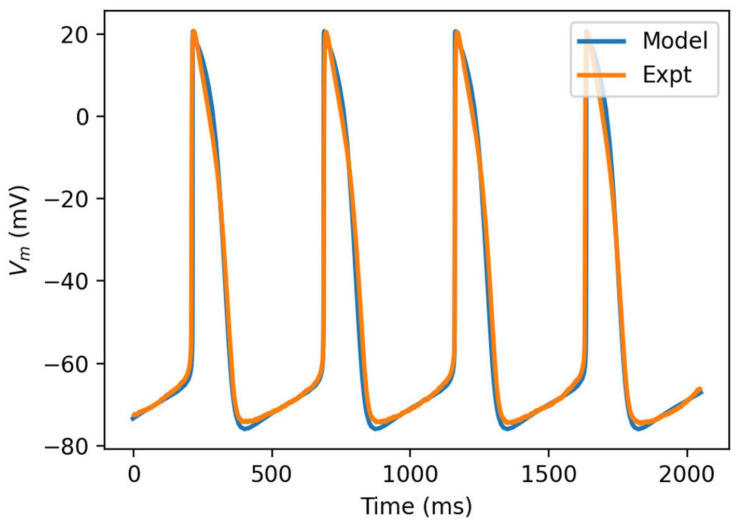
Comparison of simulated action potential morphology (blue) to experimentally recorded (orange).

**FIGURE 6:**
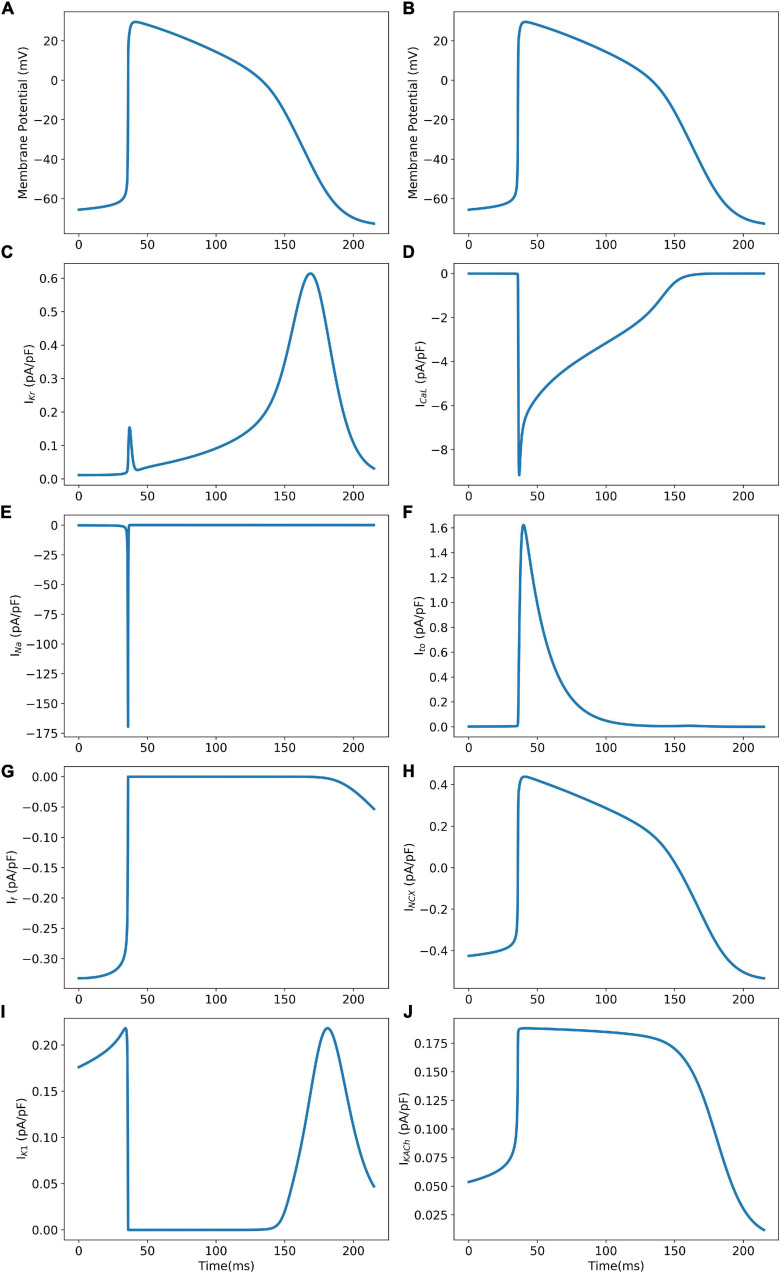
Model AP (Panels **A** and **B**) and corresponding ionic current transients (Panels **C–J**) during spontaneous AP generation.

**FIGURE 7:**
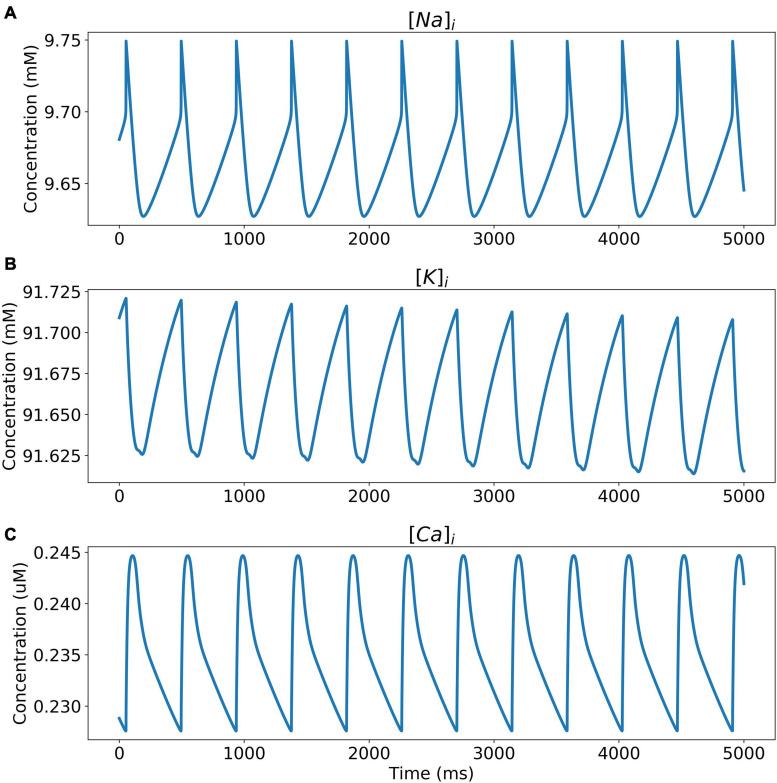
Intracellular ionic concentrations. **(A)** [Na^+^]_*i*_, **(B)** [K^+^]_*i*_, and **(C)** [Ca^2+^]_*i*_ during spontaneous APs.

**FIGURE 8:**
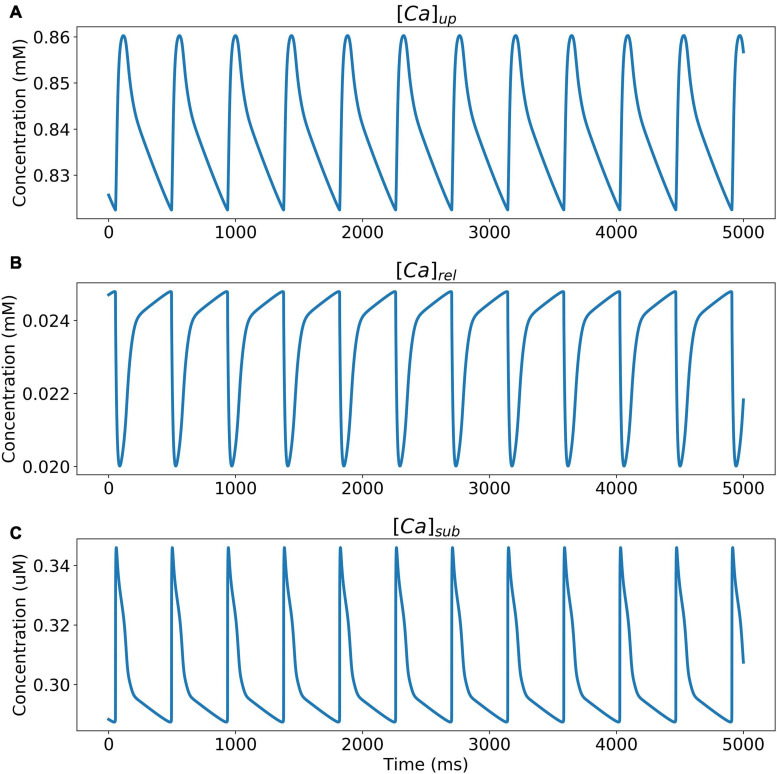
Ca^2+^ concentrations in **(A)** NSR ([Ca^2+^]_*up*_), **(B)** JSR ([Ca^2+^]_*rel*_), and **(C)** subspace ([Ca^2+^]_*sub*_) during spontaneous APs.

The model was simulated for 10 min to achieve the steady states for all currents, fluxes, and ionic concentrations. The steady state (initial conditions) values of the model variables have been reported in Supplementary Table 9. Figure 5 shows a comparison of the spontaneous APs simulated by the hiPSC-CM model as compared to the experimentally recorded ones. Our model was able to accurately reproduce the experimentally observed AP morphology as well as the automaticity of hiPSC-CMs. Time course metrics, such as the AP duration at 50, 75, and 90% repolarization, that is, APD_50_, APD_75_, and APD_90_, respectively; cycle length (CL), that is, AP peak-to-peak duration which includes the diastolic resting phase duration; maximum diastolic potential (MDP); and beats per minute (BPM), were used to assess the model AP morphology compared to the experimental counterpart. Table 4 presents the comparison of the AP metrics produced by the model to those recorded in the experiments, whereas Table 5 lists the calcium transient parameters, such as rise time from 10 to 50% and 10 to 90% of peak value (RT_1050_ and RT_1090_, respectively), decay time from 90 to 10% of peak value (DT_9010_), peak time (*T*_peak_), and frequency of spontaneous activation. Supplementary Table 10 compares the AP metrics and calcium transients of our model to those of Paci et al. (2018) and Kernik et al. (2019). It should be noted that the experimental AP traces used in our model differ considerably than those used in the other models. Wide variability in AP morphologies and calcium transients in hiPSC-CMs has been reported in literature indicating a large population heterogeneity in these cells. For example, Doss et al. (2012) documented CL variations from 327 to 7,063 ms; APD_90_ variations from 70 to 789 ms; AP amplitudes ranging from 58 to 121 mV; and *V*_max_ ranging from 5 to 86 V/s among the hiPSC-CM populations. Figure 6 shows the spontaneous AP generation and corresponding transient plots of the constituent ionic currents. Figure 7 shows the intracellular ionic concentrations ([Na^+^]_*i*_, [K^+^]_*i*_, and [Ca^2+^]_*i*_), whereas Figure 8 shows the Ca^2+^ concentrations in NSR ([Ca^2+^]_*up*_), JSR ([Ca^2+^]_*rel*_), and subspace ([Ca^2+^]_*sub*_) during spontaneous APs. Supplementary Figure 8 shows various transient ionic currents during multiple spontaneous AP firing. Supplementary Figure 9 presents a comparison of spontaneous APs vs. stimulus-elicited APs and the corresponding calcium transients in our model. The stimulus-elicited APs have a steeper Phase 0 depolarization and higher AP magnitude due to a higher *I*_Na_ amplitude. The diastolic depolarization seen in spontaneous APs is absent in paced APs due to a partial block of *I*_f_. However, the amplitude of calcium transients is higher in spontaneous APs than the paced APs.

**TABLE 4:** Comparison of action potential parameters.

Parameter	Model	Experiments*
AP duration, APD_50_ (ms)	104.93	102.596 ± 2.615
AP duration, APD_75_ (ms)	126.26	126.104 ± 2.667
AP duration, APD_90_ (ms)	142.86	141.169 ± 3.231
Cycle Length, CL (ms)	470.23	482.918 ± 21.572
Maximum diastolic potential, MDP (mV)	–75.90	–74.751 ± 0.368
Beats per minute, BPM	126.0	122.14 ± 7.73

*n = 62 APs over 29,700 ms. For BPM, 2,000 ms time window were slid over 28,000 ms total time span (i.e., *n* = 14), to obtain information on variance.

**TABLE 5:** Intracellular calcium transient parameters in our model during spontaneous AP.

Parameter	Model
Rise time from 10 to 50% of peak value (RT_1050_; ms)	7.45
Rise time from 10 to 90% of peak value (RT_1090_; ms)	30.7
Decay time from 90 to 10% of peak value (DT_9010_; ms)	127.25
Freq of spontaneous activation (Hz)	2.26
Time to peak (T_peak_; ms)	65.6

### Sensitivity Analysis

**FIGURE 9:**
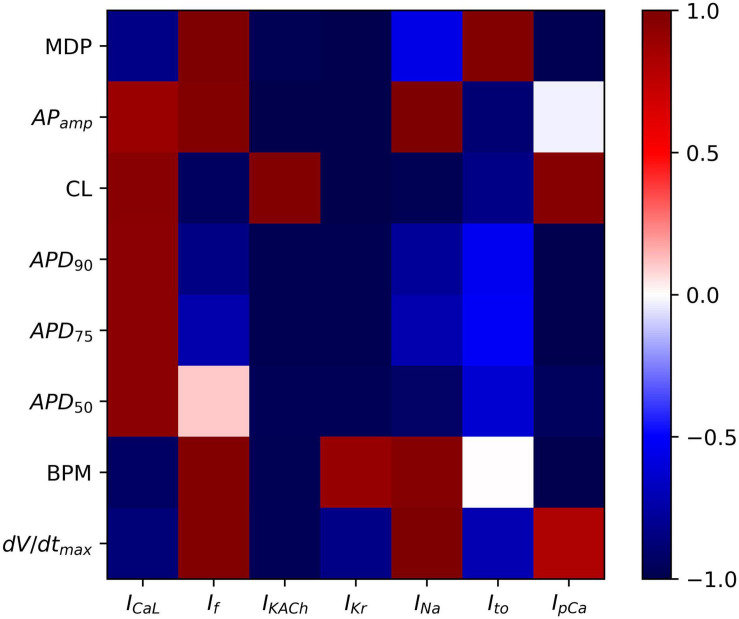
Sensitivity analysis of model showing color-coded correlation coefficients corresponding to the ionic current variations and their effects on the AP parameters.

To analyze the sensitivity of the baseline hiPSC-CM model, the maximum conductances of the various ionic currents were varied from 0 (complete block) to 200% (2 × enhancement), and their effects on the AP parameters such as APD (APD_50_, APD_75_, and APD_90_), AP magnitude, dV/dt_max_, MDP, CL, and spontaneous firing rate (BPM) were studied. Scaling factors from 0 to 200% of the baseline channel conductance values were used in computing the correlation coefficients between various AP parameters and the corresponding ionic current alterations. Figure 9 shows the outcome of the systematic sensitivity analysis of the model. The figure shows a strong positive correlation coefficient between MDP and the ionic currents *I*_f_ and *I*_to_, which indicates that an increase in either of these currents causes an increase in the MDP. *I*_KACh_, on the other hand, shows a strong negative correlation with MDP, while showing a positive correlation with CL. *I*_Kr_ expectedly shows a strong negative correlation with APD (APD_90_, APD_75_, and APD_50_). This also has implications on CL, which is revealed by a strong negative correlation. Varying the *I*_Na_ reveals its strong effect on the AP amplitude corroborated by a strong positive correlation coefficient. It, however, shows an almost neutral correlation with APD. *I*_f_ shows a strong positive correlation with MDP, implying a direct relationship between the two. A similar relationship exists between *I*_f_ and BPM. *I*_f_ also has a strong negative correlation with CL. The *I*_KACh_ shows a strong negative correlation with MDP and APD while showing a positive correlation with CL.

**FIGURE 10:**
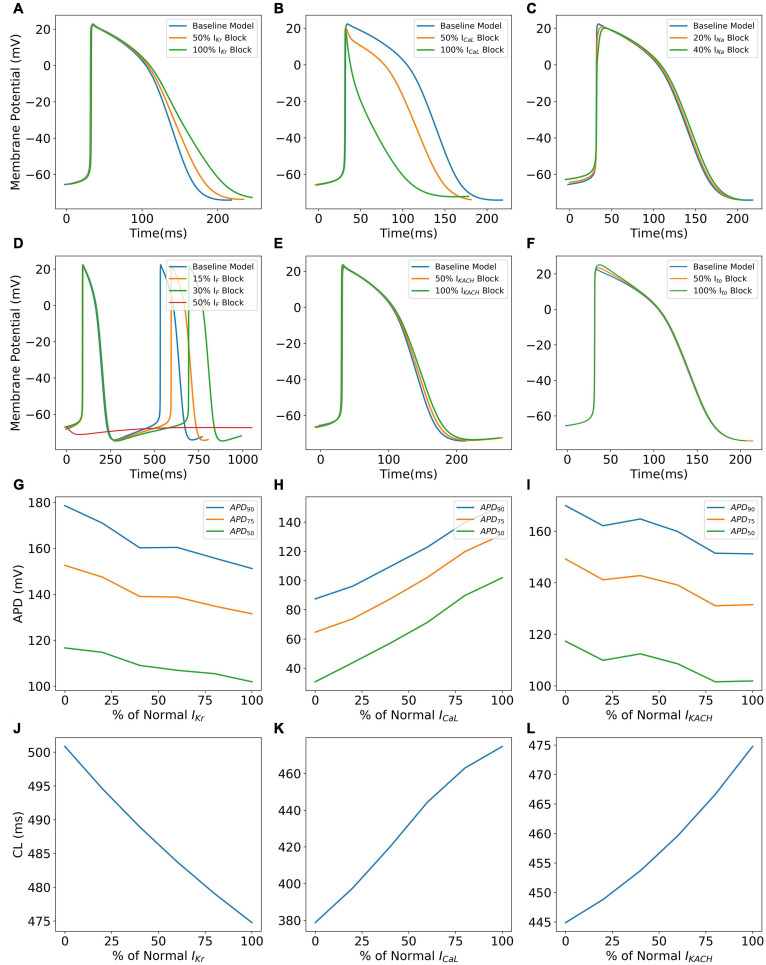
Effect of blocking various ionic currents on AP characteristics in the hiPSC-CM model such as AP morphology **(A–F)**, AP durations **(G–I)**, and cycle lengths **(J–L)**.

Figure 10 shows specific cases of ionic current blockades and their effects on the AP morphology (Figures 10A–F), AP durations (Figures 10G–I), and CL (Figures 10J–L). *I*_Kr_ is the principal repolarization current in the rapid repolarization phase in hiPSC-CMs and its blockade prolongs the AP in the Phase 3 as shown in Panel A. With a varying extent of the *I*_Kr_ block, APD successively increases (Figure 10G) and cycle length decreases (Figure 10J). Blocking *I*_CaL_ significantly shortens the AP (Figures 10B,H), thereby reducing the CL (Figure 10K). Figure 10C shows the reduction in the AP magnitude as a result of the *I*_Na_ block. Excessive blockage of *I*_Na_ (beyond 40%), however, prevents the initiation of a spontaneous AP. The pacemaker current, *I*_f_, plays a major role in maintaining spontaneous activity. A slight block in this current reduces the frequency of automaticity, and lowers the MDP and magnitude of AP (Figure 10D). More severe blocks (beyond 50%) of *I*_f_ inhibit the spontaneous AP generation. Blocking *I*_KACh_ prolongs the AP in Phases 2 and 3 as shown in Figures 10E,I. Subsequently, there is an elevation of MDP (not shown) with a higher extent of *I*_KACh_ block. Interestingly, although AP was prolonged, the CL was seen decreasing monotonically with the extent of *I*_KACh_ block (Figure 10L). It was observed that in the diastolic phase (Phase 4), a reduced repolarization effect of *I*_KACh_ favors the diastolic depolarization offered by *I*_f_.

**FIGURE 11:**
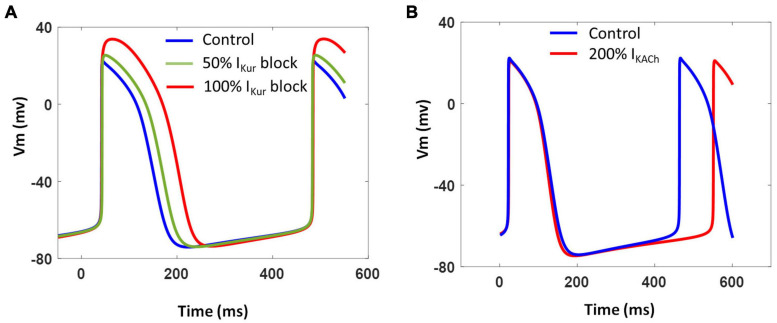
**(A)** Effect of varying extent of *I*_Kur_ block on model AP and CL. **(B)** Effect of *I*_KACh_ enhancement on model AP and CL.

We further investigated the contribution of two atria-specific currents, namely, *I*_Kur_ and *I*_KACh_, which have been recorded in atrial- and nodal-like hiPSC-CMs (Lodrini et al., 2020). Blocking *I*_Kur_ as a result of simulating the effects of 4-AP (50 μM) reduced Phase 1 repolarization resulting in AP prolongation and increased AP magnitude as shown in Figure 11A. The extent of AP prolongation was proportional to the extent of *I*_Kur_ block. The spontaneous firing rate (BPM), however, remained unchanged. The AP prolongation in all phases of repolarization in our model is in agreement with the recent experimental findings (Devalla et al., 2015). Figure 11B shows the effect of vagal stimulation by carbachol (CCh; 10 μM). The 200% enhancement of *I*_KACh_ slowed down the spontaneous activity by ∼10% without any significant effect on the APD.

**FIGURE 12:**
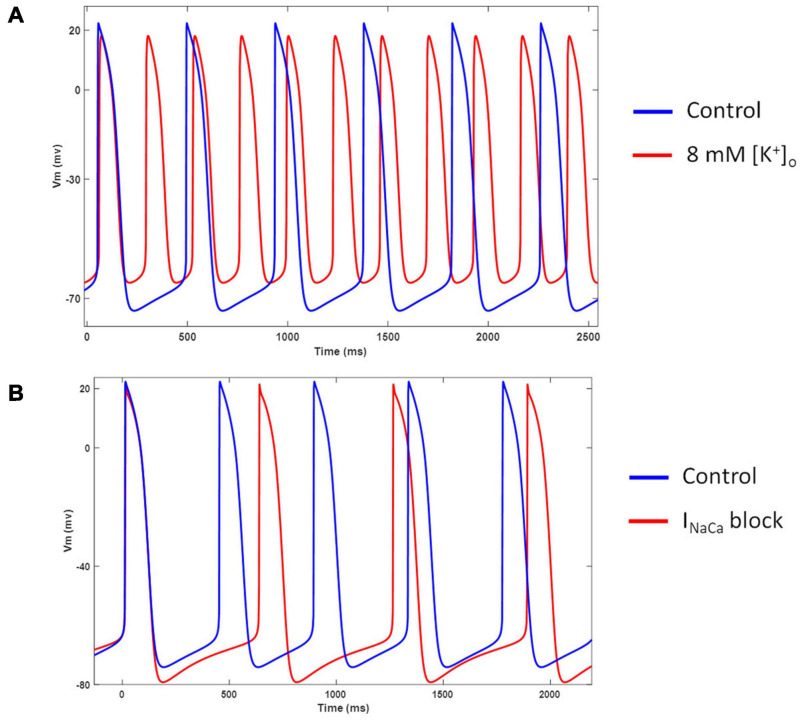
**(A)** Effect of hyperkalemia ([K^+^]_o_ = 8 mM) conditions on model AP and frequency of automaticity. **(B)** Effect of *I*_*NaCa*_ blockade on model AP and frequency of automaticity.

Figure 12A shows the model behavior in hyperkalemic conditions. Our model showed an increased spontaneous firing rate and diastolic depolarization when extracellular K^+^ was increased from 5.4 to 8 mM. This behavior is in agreement with several experimental studies in nodal cells (DiFrancesco et al., 1986; Frace et al., 1992; Hoppe and Beuckelmann, 1998). Figure 12B shows the effects of completely blocking *I*_*NaCa*_. Inhibition of *I*_*NaCa*_ reduces the spontaneous firing rate and hyperpolarizes the membrane potential in our model. However, it does not abolish the spontaneous activity as reported in (Blinova et al., 2018).

### Effects of Adrenergic and Cholinergic Stimulation

**FIGURE 13:**
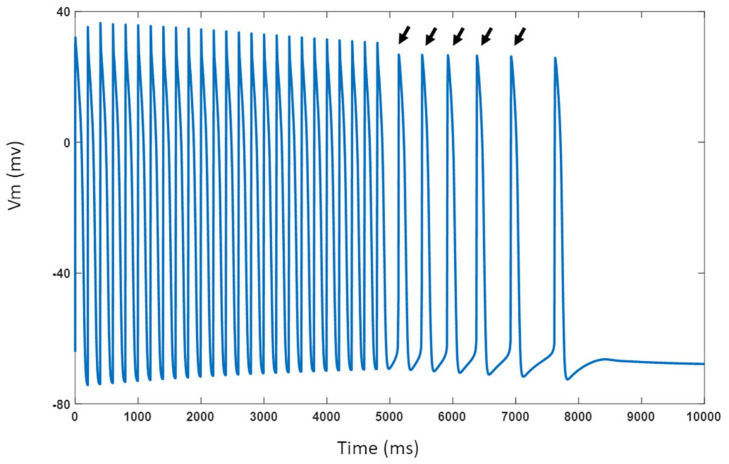
Model response to 5 Hz burst pacing in presence of isoproterenol effects. Black arrows indicate spontaneous DAD-triggered APs post burst-pacing.

**FIGURE 14:**
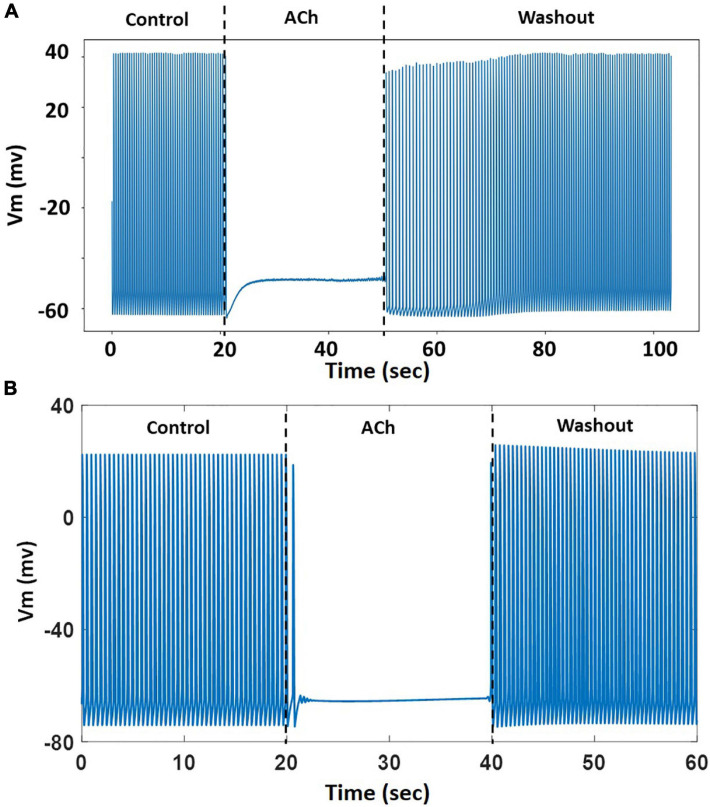
**(A)** A representative experimental AP trace in hiPSC-CM treated with 1 μM ACh. **(B)** Model behavior with 300% enhancement of *I*_KACh_ simulating the effects of ACh. The ACh was administered from 20–40 s followed by washout.

The model was challenged with stressors such as adrenergic stimulation using isoproterenol and cholinergic stimulation using ACh. For isoproterenol stimulation, the spontaneous APs were suppressed by blocking *I*_f_ by 50%. The model was burst paced at 5 Hz for 5 s to overload the SR with Ca^2+^ in the presence of isoproterenol effects. The model exhibited several spontaneous DAD-triggered APs post burst-pacing as shown in Figure 13 (black arrows). Figure 14A shows experimental AP traces when the spontaneously beating hiPSC-CMs were treated with 1 μM ACh. The ACh was administered for 20–40 s which depolarized the membrane and suppressed the spontaneous AP firing. This was unexpected because one would expect the spontaneous activity to slow down similar to that in atrial cells (or as seen in Figure 11B). After the washout of ACh (post 40 s), the spontaneous AP was resumed, albeit at a slightly higher frequency. Our model was able to reproduce the experimental behavior (shown in Figure 14A) when *I*_KACh_ was enhanced to 300%, as shown in Figure 14B. Additionally, our model was able to provide useful insights into the unusual behavior observed. The ACh exposure causes a persistently increased *I*_KACh_ during diastolic interval, which balances the inward *I*_f_, thereby suppressing the AP generation. After washout, the *I*_KACh_ returned to normal levels and the *I*_f_-facilitated spontaneous APs resumed. The elevated intracellular K^+^ levels cause the frequency of spontaneous AP to be higher after washout which gradually returned to a normal firing rate.

## Discussion

We present a robust approach to fit the experimental electrophysiological data to theoretical formulations in order to develop high-fidelity numerical biophysical models. We demonstrate the effectiveness of a GA-based optimization method to develop an *in silico* model of hiPSC-CMs that accurately reproduces the experimental measurements. The model behavior was extensively validated based on AP morphology, sensitivity analysis, and various ion channel blocking mechanisms.

The experimental electrophysiological data in hiPSC-CMs show a wide range of variability, presumably due to the different techniques used to direct the hiPSCs to the cardiac lineage (Biendarra-Tiegs et al., 2020). This imposes additional challenges in fitting the experimental data to model equations. We utilized the GA method to attain model optimization, which is an evolutionary metaheuristic method inspired by Darwinian evolution and natural selection. Optimization, by this approach, is executed in a stochastic combinatorial manner which makes it less susceptible to getting stuck at local minima, unlike the gradient-based methods, and tend to converge at the global optimized solution (Smith et al., 1995). Previous attempts at implementing GA-based fitting of cardiac models mostly focused on the simple fitting of maximum channel conductance to recapitulate the desired AP morphology (Syed et al., 2005; Bot et al., 2012; Groenendaal et al., 2015; Tomek et al., 2019; Smirnov et al., 2020). We, on the other hand, performed GA optimization at the underlying ionic current level, which is a more realistic and robust approach. Our approach ensures that the model ionic formulations adhere correctly to the experimental recordings and is especially more suitable for modeling hiPSC cells which exhibit a wide range of phenomenological variation. Smirnov et al. (2020) modified the GA protocol by Bot et al. (2012) by adopting vector mutation terms from the Cauchy distribution that promote drastic variance between the mutants and their uncorrelated parent proportions. Tomek et al. (2019), on the other hand, used a multi-objective GA approach for parameter fitting. These works, however, do not mention constraining the range of each gene to a defined neighborhood of the respective original model parameters. Since the uniqueness of a solution is often not guaranteed in such non-convex optimization problems, physiological relevance must be upheld. We address this by imposing the customizable constraint, λ_*a*_, which symmetrically bounds the range from which the initial parameter values (from published physiologically validated models) are drawn. The significance of this constraint, in conjunction with the population size, is the ability to define the initial search resolution. A wider unconstrained parameter range is likely to be initially underrepresented if the population size is not large enough, which, in turn, may favor the speed of convergence (offered by extreme chromosomal variance) over physiological relevance. Another novelty in our GA protocol, to the best of our knowledge, is the correlation between the parent proportion and the number of crossover points. To maintain sufficient diversity, more offspring and mutants are produced as the number of crossover points increase. Furthermore, an increase in the crossover points offer an extensive combinatorial gene shuffling during crossover per generation; we therefore propose a proportionate increase in the population size to accommodate the resulting diverse offspring and mutants.

There have been very few attempts at implementing hiPSC-CM biophysical models due to inconsistent experimental data. An earlier model by Paci et al. (2013) was formulated based on the limited data at that time. More recent models by Paci et al. (2018, 2020), and Koivumäki et al. (2018) incorporated a more realistic calcium handling. The study by Kernik et al. (2019) adopted experimental data from multiple sources in an attempt to cover the range of variability seen in these cells. However, the unresolved inconsistencies in the recording and clamping protocols used by the various sources have the potential to introduce unwarranted deviations in the hiPSC-CM electrophysiological parameter range as well as the generalizability of the baseline model. Our model recapitulates the experimentally recorded hiPSC-CM AP morphology with high fidelity. Moreover, it is able to qualitatively reproduce the experimentally reported (Ma et al., 2011) effects of: (i) APD prolongation caused by *I*_Kr_ block, (ii) reduction in AP magnitude and rate of change of upstroke voltage as a result of Na^+^ channel block, (iii) loss of notch-shaped AP (Phase 1 repolarization) due to *I*_to_ blockade, (iv) triangulation of AP due to *I*_Kr_ or *I*_to_ blockades, (v) significant AP shortening due to *I*_CaL_ block, (vi) loss of automaticity as a result of *I*_f_ blockade, and (vii) alterations in spontaneous firing rate as a result of I_*NCX*_ block and hyperkalemic conditions. Our model was also able to produce the experimentally observed effects of channel blocks on the frequency of spontaneous APs (cycle length) and MDPs in hiPSC cells. In the presence of adrenergic stimulation and hypercalcemia, our model was able to generate a DAD-induced triggered activity as a result of SR Ca^2+^ overload.

One of the advantages of our model over the existing ones is the inclusion of atria-specific ionic channels, *I*_Kur_ and *I*_KACh_, which have recently been found to be present and functional in hiPSC-CMs (Zhao et al., 2018). The inclusion of *I*_KACh_ allows for the investigation of the variability in the spontaneous beating frequency influenced by parasympathetic influences and/or the presence of ACh which have been found to reduce the heart rate. Indeed, our model was able to reproduce and provide explanation on the experimentally observed suppression of automaticity in hiPSC-CMs upon treatment with ACh. Moreover, our model can qualitatively reproduce the effects of atria-specific drugs such as carbachol and 4-AP. *I*_KACh_ is actively involved in the maintenance of atrial fibrillation, including chronic atrial fibrillation (Dobrev et al., 2005). Recent advances in cell differentiation techniques use *I*_KACh_ and *I*_Kur_ as markers to identify atrial-like hiPSC-CMs, which were preferentially produced by retinoic acid treatment (Argenziano et al., 2018). The atrial-like CMs are being increasingly used for disease modeling and pre-clinical screening of antiarrhythmic drugs (Devalla et al., 2015). As such, our model is valuable in disease modeling and simulations of atrial phenotypes.

We adopted simpler Hodgkin-Huxley type current formulations to limit the number of optimization parameters and to keep the simulations computationally tractable. It also avoided overfitting of the experimental data. The main aim of this study was to demonstrate the feasibility of GA-based parametrization of model equations which was done by optimizing five ionic current formulations based on the experimental data. The remaining components of the model, including the intracellular calcium handling, were adopted from a nodal cell model, which has a very close morphological resemblance with the hiPSC-CMs. Nonetheless, the GA-based optimization can be seamlessly extended to the whole cell level as more and more experimental data becomes available. The calcium handling in our model is based on nodal cell formulations and may not represent true calcium transients in hiPSC-CMs. Our model is able to reproduce DAD-triggered APs, but does not produce other arrhythmogenic events such as EADs and alternans. Our model also does not reproduce the hyperkalemia-induced slowing of spontaneous activity as shown by Blinova et al. (2018). The hiPSC-CM population used in Blinova et al. (2018) showed a minor role of *I*_f_ in sustaining the spontaneous electrical activity, as revealed by their tests with ivabradine [see the original Figure 2A(i)], which could explain the different response of hiPSC-CMs to hyperkalemia reported in their study. Notwithstanding these limitations, our model behavior is consistent with the findings of various *in vitro* drug tests, in which such arrhythmic markers were observed only in a portion of the pluripotent cells used for testing (Blinova et al., 2018).

## Conclusion

Human induced pluripotent stem cell-derived cardiomyocytes have received significant attention lately; with applications in regenerative medicine, cardiac safety pharmacology, and the implementation of patient specific models for studying drug-induced and inherited cardiac diseases. We present a GA-based model parametrization methodology to incorporate experimental data into numerical models. The proposed method was utilized to formulate a biophysical computer model of hiPSC-CMs based on the experimental data and available literature, as a potential tool for studying and simulating the dynamics of hiPSC-CM electrophysiology. Ionic current formulations of five key currents, namely, fast sodium current (*I*_Na_), transient outward potassium current (*I*_to_), L-type calcium current (*I*_CaL_), rapid delayed rectifier current (*I*_Kr_), and hyperpolarization-activated current (*I*_f_), were optimized by the GA protocol to fit their experimental data. These were then combined with adjusted formulations for nine other currents imported from existing models to faithfully reproduce experimentally obtained hiPSC-CM AP morphology and spontaneous activity. The model was able to accurately reproduce the experimentally recorded AP characteristics and channel blocking drug effects. The model was able to provide insights into the causes of the experimentally observed suppression of automaticity in hiPSC-CMs during cholinergic stimulation. The outcome of this work has implications on validating the biophysics of hiPSC-CMs in their use as viable substitutes for human cardiomyocytes. Specifically, in the study of inherited cardiac disorders and in cardiac safety pharmacology, where drug-induced cardiac disorders are investigated.

## Data Availability Statement

The raw data supporting the conclusions of this article will be made available by the authors, without undue reservation.

## Author Contributions

AA performed the model formulations, GA-based parameterization, and model simulations. BT performed the single-cell simulations and sensitivity analysis. PY performed the model assessment and data analysis. JT and JC performed the electrophysiology experiments and experimental data analysis. JC also provided experimental and clinical guidance wherever needed. MB-H reviewed and modified the mathematical formulations. MD conceived the study, assessed the model, guided the students, and confirmed the model outcomes. All authors contributed to the article and approved the submitted version.

## Conflict of Interest

The authors declare that the research was conducted in the absence of any commercial or financial relationships that could be construed as a potential conflict of interest.
