# Supplementary material for: An in silico hiPSC-Derived Cardiomyocyte Model Built With Genetic Algorithm
Source: Front Physiol. 2021 Jun 16;12:675867. doi: 10.3389/fphys.2021.675867 (PMC8242263; doi:10.3389/fphys.2021.675867)
Supplement: Supplementary file 1 [file Data_Sheet_1.PDF]

Supplementary Material

An *In Silico* hiPSC-derived Cardiomyocyte Model Built with Genetic Algorithm

A. D. Akwaboah, B. Tsevi, P. Yamlome, J. A. Treat, M. Brucal-Hallare, J. M. Cordeiro, M. Deo*

Code Availability

Model implementation available at:

https://github.com/Adakwaboah/hiPSC-CM_Computational_Model

1. I_{Na} Parametrization (20 free parameters)

$p_{I_{Na}}: \{\boldsymbol{p_{I_{Na}}}[\mathbf{0}],\ \boldsymbol{p_{I_{Na}}}[\mathbf{1}],\ \boldsymbol{p_{I_{Na}}}[\mathbf{2}],\ \cdots,\ \boldsymbol{p_{I_{Na}}}[\mathbf{19}]\}$

$$a_m = \frac{\boldsymbol{p_{I_{Na}}}[\mathbf{1}] \cdot (V_m + 47.13)}{1 - exp\left(-0.1 \cdot (V_m + 47.13)\right)} \tag{1}$$

$$b_m = \boldsymbol{p_{I_{Na}}}[\mathbf{2}] \cdot exp\left(\frac{-V_m}{11}\right) \tag{2}$$

$$\tau_m = \frac{1}{a_m + b_m} \tag{3}$$

$$m_\infty = a_m \cdot \tau_m \tag{4}$$

$$a_h = \boldsymbol{p_{I_{Na}}}[\mathbf{3}] \cdot exp\left(\frac{V_m + 80}{-6.8}\right), V_m < -40mV \tag{5}$$

$$a_h = 0, otherwise$$

$$b_h = \boldsymbol{p_{I_{Na}}}[\mathbf{4}] \cdot exp(0.079 \cdot V_m) + \boldsymbol{p_{I_{Na}}}[\mathbf{5}] \cdot exp(0.35 \cdot V_m), V_m < -40mV \tag{6}$$

$$b_h = \frac{1}{\boldsymbol{p_{I_{Na}}}[\mathbf{13}] \cdot \left(-\frac{V_m + \boldsymbol{p_{I_{Na}}}[\mathbf{14}]}{\boldsymbol{p_{I_{Na}}}[\mathbf{15}]}\right)} \quad , otherwise$$

$$\tau_h = \frac{1}{a_h + b_h} \tag{7}$$

$$h_\infty = a_h \cdot \tau_h \tag{8}$$

$$a_j = \frac{\left[-\boldsymbol{p_{I_{Na}}}[\mathbf{6}] \cdot exp\left(\boldsymbol{p_{I_{Na}}}[\mathbf{7}] \cdot V_m\right) - \boldsymbol{p_{I_{Na}}}[\mathbf{8}] \cdot exp\left(-\boldsymbol{p_{I_{Na}}}[\mathbf{9}] \cdot V_m\right)\right](V_m + 37.78)}{1 + exp\left(\boldsymbol{p_{I_{Na}}}[\mathbf{10}] \cdot (V_m + 79.23)\right)} \tag{9}$$

$$, V_m < -40mV$$

$$a_j = 0, \quad otherwise$$

$$b_j = \frac{\boldsymbol{p_{I_{Na}}}[\mathbf{11}] \cdot exp(-\boldsymbol{p_{I_{Na}}}[\mathbf{12}] \cdot V_m)}{1 + exp(-0.1378 \cdot (V_m + 40.14))}, V_m < -40mV \tag{10}$$

$$b_j = \frac{\boldsymbol{p_{I_{Na}}}[\mathbf{16}] \cdot exp(-\boldsymbol{p_{I_{Na}}}[\mathbf{17}] \cdot V_m)}{1 + exp\left(-\boldsymbol{p_{I_{Na}}}[\mathbf{18}] \cdot \left(V_m + \boldsymbol{p_{I_{Na}}}[\mathbf{19}]\right)\right)}, otherwise$$

$$\tau_j = \frac{1}{a_j + b_j} \tag{11}$$

$$j_\infty = a_j \cdot \tau_j \tag{12}$$

$$\frac{dm}{dt} = \frac{m_\infty - m}{\tau_m} \tag{13}$$

$$\frac{dh}{dt} = \frac{h_\infty - h}{\tau_h} \tag{14}$$

$$\frac{dj}{dt} = \frac{j_\infty - j}{\tau_j} \tag{15}$$

$$I_{Na} = \boldsymbol{p_{I_{Na}}}[\mathbf{0}] \cdot m^3 \cdot h \cdot j \cdot (V_m - E_{Na}) \tag{16}$$

Table S1. Initial and fitted parameter values I_{Na} formulation.

**Parameter**	$\boldsymbol{p_{I_{Na}}}[\mathbf{0}]$	$\boldsymbol{p_{I_{Na}}}[\mathbf{1}]$	$\boldsymbol{p_{I_{Na}}}[\mathbf{2}]$	$\boldsymbol{p_{I_{Na}}}[\mathbf{3}]$	$\boldsymbol{p_{I_{Na}}}[\mathbf{4}]$	$\boldsymbol{p_{I_{Na}}}[\mathbf{5}]$	$\boldsymbol{p_{I_{Na}}}[\mathbf{6}]$
Original	18 [1]	0.32	0.08	0.135	3.56	310000	127140
Fitted (Rep.)	10.18724	0.281165	0.041103	0.185913	2.335591	269343.1	94426.26
% Change	-43.40	-12.14	-48.62	37.71	-34.39	-13.12	-25.73
**Parameter**	$\boldsymbol{p_{I_{Na}}}[\mathbf{7}]$	$\boldsymbol{p_{I_{Na}}}[\mathbf{8}]$	$\boldsymbol{p_{I_{Na}}}[\mathbf{9}]$	$\boldsymbol{p_{I_{Na}}}[\mathbf{10}]$	$\boldsymbol{p_{I_{Na}}}[\mathbf{11}]$	$\boldsymbol{p_{I_{Na}}}[\mathbf{12}]$	$\boldsymbol{p_{I_{Na}}}[\mathbf{13}]$
Original	0.2444	3.47e-05	0.04391	0.311	0.1212	0.01052	0.13
Fitted (Rep.)	0.195789	4.34e-05	0.059582	0.429536	0.126055	0.005454	0.111373
% Change	-19.89	25.07	35.70	38.114	4.01	-48.16	-14.33
**Parameter**	$\boldsymbol{p_{I_{Na}}}[\mathbf{14}]$	$\boldsymbol{p_{I_{Na}}}[\mathbf{15}]$	$\boldsymbol{p_{I_{Na}}}[\mathbf{16}]$	$\boldsymbol{p_{I_{Na}}}[\mathbf{17}]$	$\boldsymbol{p_{I_{Na}}}[\mathbf{18}]$	$\boldsymbol{p_{I_{Na}}}[\mathbf{19}]$	
Original	10.66	11.1	0.3	2.54e-07	0.1	32	
Fitted (Rep.)	0.823823	7.983436	0.43717	2.87e-07	0.024221	52.26473	
% Change	-92.27	-28.08	45.72	13.00	-75.78	63.33	

[1] The original value for $\boldsymbol{p_{I_{Na}}}[\mathbf{0}]$ is 16 in the LRII model. This was adjusted to qualitatively match the model and experimental peak values prior to fitting. The motivation for this was to accelerate the parameter search time due to a reduced initial variance between model output and experimental data.

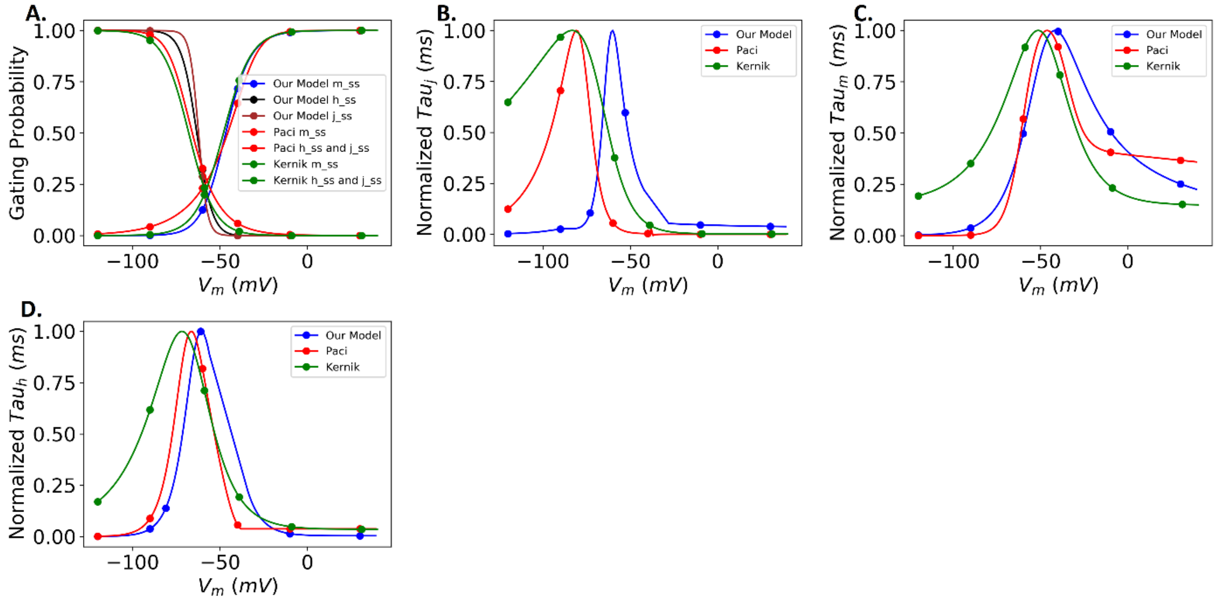

Figure S1. Comparison of I_{Na} activation, inactivation and corresponding time constants in our model with those of Paci et al. [1] (Paci2018 model) and Kernik et al. [2]

2. I_{Kr} Parametrization (18 free parameters)

$$\boldsymbol{p_{I_{Kr}}}: \{ \boldsymbol{p_{I_{Kr}}[0]}, \boldsymbol{p_{I_{Kr}}[1]}, \boldsymbol{p_{I_{Kr}}[2]}, \cdots, \boldsymbol{p_{I_{Kr}}[17]} \}$$

$$p_{a_\infty} = \frac{1}{1 + exp\left(-\frac{V_m + \boldsymbol{p_{I_{Kr}}[1]}}{\boldsymbol{p_{I_{Kr}}[2]}}\right)} \tag{17}$$

$$p_{i_\infty} = \frac{1}{1 + exp\left(\frac{V_m + \boldsymbol{p_{I_{Kr}}[3]}}{\boldsymbol{p_{I_{Kr}}[4]}}\right)} \tag{18}$$

$$\tau_{p_{aF}} = \frac{\boldsymbol{p_{I_{Kr}}[5]}}{\boldsymbol{p_{I_{Kr}}[6]} \cdot exp\left(\frac{V_m}{\boldsymbol{p_{I_{Kr}}[7]}}\right) + \boldsymbol{p_{I_{Kr}}[8]} \cdot \boldsymbol{exp}\left(-\frac{\boldsymbol{V_m}}{\boldsymbol{p_{I_{Kr}}[9]}}\right)} \tag{19}$$

$$\tau_{p_{aS}} = \frac{\boldsymbol{p_{I_{Kr}}[5]}}{\boldsymbol{p_{I_{Kr}}[10]} \cdot exp\left(\frac{V_m}{\boldsymbol{p_{I_{Kr}}[11]}}\right) + \boldsymbol{p_{I_{Kr}}[12]} \cdot \boldsymbol{exp}\left(-\frac{\boldsymbol{V_m}}{\boldsymbol{p_{I_{Kr}}[13]}}\right)} \tag{20}$$

$$\tau_{p_i} = \frac{1}{\boldsymbol{p_{I_{Kr}}[14]} \cdot exp\left(-\frac{V_m}{\boldsymbol{p_{I_{Kr}}[15]}}\right) + \boldsymbol{p_{I_{Kr}}[16]} \cdot \boldsymbol{exp}\left(\frac{\boldsymbol{V_m}}{\boldsymbol{p_{I_{Kr}}[17]}}\right)} \tag{21}$$

$$\frac{dp_{aF}}{dt} = \frac{p_{a_\infty} - p_{aF}}{\tau_{p_{aF}}} \tag{22}$$

$$\frac{dp_{aS}}{dt} = \frac{p_{a_\infty} - p_{aS}}{\tau_{p_{aS}}} \tag{23}$$

$$\frac{dp_i}{dt} = \frac{p_{i_\infty} - p_i}{\tau_{p_i}} \quad (24)$$

$$I_{Kr} = \boldsymbol{p}_{\boldsymbol{I_{Kr}}}[\mathbf{0}] \cdot (V_m - E_K) \cdot (0.6 \cdot p_{aF} + 0.4 \cdot p_{aS}) \cdot p_i \quad (25)$$

Table S2. Initial and fitted parameter values I_{Kr} formulation.

**Parameter**	$\boldsymbol{p_{I_{Kr}}}[\mathbf{0}]$	$\boldsymbol{p_{I_{Kr}}}[\mathbf{1}]$	$\boldsymbol{p_{I_{Kr}}}[\mathbf{2}]$	$\boldsymbol{p_{I_{Kr}}}[\mathbf{3}]$	$\boldsymbol{p_{I_{Kr}}}[\mathbf{4}]$	$\boldsymbol{p_{I_{Kr}}}[\mathbf{5}]$
Original	0.02	23.2	10.6	28.6	17.1	0.84655
Fitted (Rep.)	0.01865	6.42653	11.87998	40.11632	14.08222	9.63757
% Change	-6.75	-72.30	12.08	40.27	-17.65	1038.45
**Parameter**	$\boldsymbol{p_{I_{Kr}}}[\mathbf{6}]$	$\boldsymbol{p_{I_{Kr}}}[\mathbf{7}]$	$\boldsymbol{p_{I_{Kr}}}[\mathbf{8}]$	$\boldsymbol{p_{I_{Kr}}}[\mathbf{9}]$	$\boldsymbol{p_{I_{Kr}}}[\mathbf{10}]$	$\boldsymbol{p_{I_{Kr}}}[\mathbf{11}]$
Original	0.0372	15.9	0.00096	22.5	0.0042	17
Fitted (Rep.)	0.04237	17.03561	0.00124	23.34742	0.00457	14.61857
% Change	13.90	7.14	29.17	3.77	8.81	-14.01
**Parameter**	$\boldsymbol{p_{I_{Kr}}}[\mathbf{12}]$	$\boldsymbol{p_{I_{Kr}}}[\mathbf{13}]$	$\boldsymbol{p_{I_{Kr}}}[\mathbf{14}]$	$\boldsymbol{p_{I_{Kr}}}[\mathbf{15}]$	$\boldsymbol{p_{I_{Kr}}}[\mathbf{16}]$	$\boldsymbol{p_{I_{Kr}}}[\mathbf{17}]$
Original	0.00015	21.6	0.1	54.645	0.656	106.157
Fitted (Rep.)	0.000172	17.37150	0.08471	52.15060	0.75996	107.97715
% Change	14.67	-19.58	-15.29	-4.56	15.85	1.71

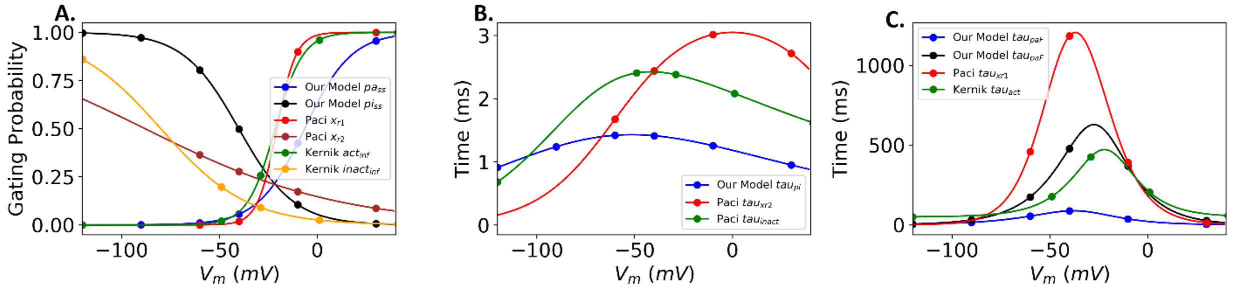

Figure S2. Comparison of I_{Kr} activation, inactivation and corresponding time constants in our model with those of Paci et al. [1] (Paci2018 model) and Kernik et al. [2]

3. I_{to} Parametrization (20 free parameters)

Prior to parametrizing, the steady state inactivation $V_{\frac{1}{2}} = 41.1mV$ and slope, $k = 6.68$ reported by Cordeiro *et al*. [3] are applied on y_{to_∞}.

$$\boldsymbol{p}_{\boldsymbol{I_{to}}}: \{\boldsymbol{p}_{\boldsymbol{I_{to}}}[\mathbf{0}], \boldsymbol{p}_{\boldsymbol{I_{to}}}[\mathbf{1}], \boldsymbol{p}_{\boldsymbol{I_{to}}}[\mathbf{2}], \cdots, \boldsymbol{p}_{\boldsymbol{I_{to}}}[\mathbf{19}]\}$$

$$x_{to_\infty} = \frac{1}{1 + exp\left(-\frac{V_m - \boldsymbol{p}_{\boldsymbol{I_{to}}}[\mathbf{2}]}{\boldsymbol{p}_{\boldsymbol{I_{to}}}[\mathbf{3}]}\right)} \quad (26)$$

$$y_{to\infty} = \frac{1}{1 + exp\left(\frac{V_m + 41.1}{6.68}\right)} \tag{27}$$

$$\tau_{xto_f} = \boldsymbol{p_{I_{to}}}[\mathbf{4}] \cdot exp\left(-\left(\frac{V_m + \boldsymbol{p_{I_{to}}}[\mathbf{5}]}{\boldsymbol{p_{I_{to}}}[\mathbf{6}]}\right)^2\right) + \boldsymbol{p_{I_{to}}}[\mathbf{7}] \tag{28}$$

$$\tau_{yto_f} = \boldsymbol{p_{I_{to}}}[\mathbf{8}] \cdot exp\left(-\frac{\left(V_m + \boldsymbol{p_{I_{to}}}[\mathbf{9}]\right)^2}{\boldsymbol{p_{I_{to}}}[\mathbf{10}]}\right) + \boldsymbol{p_{I_{to}}}[\mathbf{11}] \tag{29}$$

$$\frac{dx_{to_f}}{dt} = \frac{x_{to\infty} - x_{to_f}}{\tau_{xto_f}} \tag{30}$$

$$\frac{dy_{to_f}}{dt} = \frac{y_{to\infty} - y_{to_f}}{\tau_{yto_f}} \tag{31}$$

$$\tau_{x_{to_s}} = \frac{\boldsymbol{p_{I_{to}}}[\mathbf{12}]}{1 + exp\left(\frac{V_m + \boldsymbol{p_{I_{to}}}[\mathbf{13}]}{\boldsymbol{p_{I_{to}}}[\mathbf{14}]}\right)} + \boldsymbol{p_{I_{to}}}[\mathbf{15}] \tag{32}$$

$$\tau_{y_{to_s}} = \frac{\boldsymbol{p_{I_{to}}}[\mathbf{16}]}{1 + exp\left(\frac{V_m + \boldsymbol{p_{I_{to}}}[\mathbf{17}]}{\boldsymbol{p_{I_{to}}}[\mathbf{18}]}\right)} + \boldsymbol{p_{I_{to}}}[\mathbf{19}] \tag{33}$$

$$\frac{dx_{to_s}}{dt} = \frac{x_{to\infty} - x_{to_s}}{\tau_{x_{to_s}}} \tag{34}$$

$$\frac{dy_{to_s}}{dt} = \frac{y_{to\infty} - y_{to_s}}{\tau_{y_{to_s}}} \tag{35}$$

$$I_{to_f} = \boldsymbol{p_{I_{to}}}[\mathbf{0}] \cdot x_{to_f} \cdot y_{to_f} \cdot (V_m - E_K) \tag{36}$$

$$I_{to_s} = \boldsymbol{p_{I_{to}}}[\mathbf{1}] \cdot x_{to_s} \cdot y_{to_s} \cdot (V_m - E_K) \tag{37}$$

$$I_{to} = I_{to_f} + I_{to_s} \tag{38}$$

Table S3. Initial and fitted parameter values I_{to} formulation.

Parameter	$p_{I_{to}}[0]$	$p_{I_{to}}[1]$	$p_{I_{to}}[2]$	$p_{I_{to}}[3]$	$p_{I_{to}}[4]$	$p_{I_{to}}[5]$	$p_{I_{to}}[6]$
Original	0.0014	0.0376	19	13	8.5	45	50
Fitted (Rep.)	0.001078	0.155693	14.68251	15.91006	3.831184	48.03285	48.96
% Change	-23.00	314.08	-22.72	22.39	-54.93	6.74	-2.08
**Parameter**	$p_{I_{to}}[7]$	$p_{I_{to}}[8]$	$p_{I_{to}}[9]$	$p_{I_{to}}[10]$	$p_{I_{to}}[11]$	$p_{I_{to}}[12]$	$p_{I_{to}}[13]$
Original	0.5	85	40	220	7	9	3
Fitted (Rep.)	0.285524	123.5405	50.31286	316.3192	13.28863	4.132051	2.300548
% Change	-42.90	45.34	25.78	43.78	89.84	-54.09	-23.32
**Parameter**	$p_{I_{to}}[14]$	$p_{I_{to}}[15]$	$p_{I_{to}}[16]$	$p_{I_{to}}[17]$	$p_{I_{to}}[18]$	$p_{I_{to}}[19]$	
Original	15	0.5	800	60	10	30	
Fitted (Rep.)	26.71314	0.112599	1135.962	49.58013	5.531819	19.71358	
% Change	78.09	-77.48	42.00	-17.37	-44.68	-34.29	

Activation of Ito though rapid, is relatively slower compared to I_{Na} and I_{CaL} and hence does not warrant very small time steps. Regardless, we simulated I_{to} over a span of 95 ms with times steps of 1.0 ms, 0.5 ms, 0.1ms and 0.001ms and confirmed the numerical accuracy in terms of R^2 values. Below table shows the computational parameters of these runs on a 2.4 GHz CPU.

Table S4. Numerical stability and run time of explicit solver for computing I_{to} parameterization.

dt (ms)	Stable?	R^2	Runtime (s)
1.0	No	-	-
0.5	Yes	0.9960	0.476
0.1	Yes	0.9995	2.176
0.001	Yes	0.9994	122.182

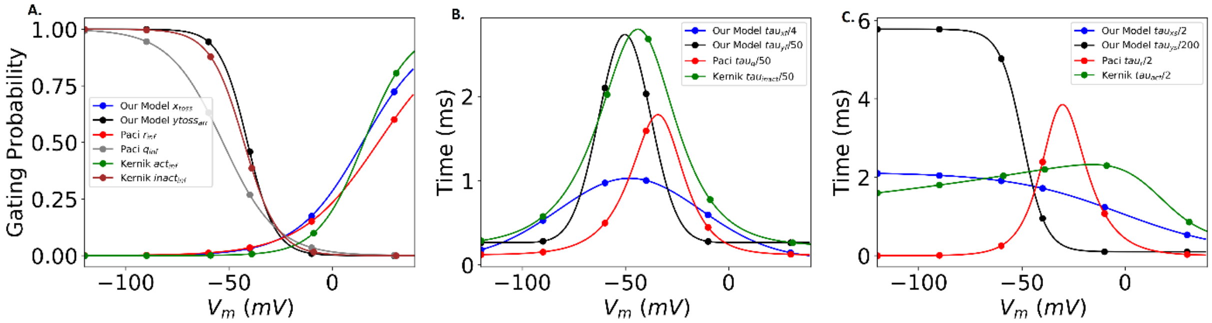

Figure S3. Comparison of I_{to} activation, inactivation and corresponding time constants in our model with those of Paci et al. [1] (Paci2018 model) and Kernik et al. [2]

4. I_{CaL} formulation (6 free parameters)

In this case, the steady state activation and inactivation parameters, i.e. half activation/ inactivation voltages and slopes, were parametrized for optimization as initial model IV plot appeared merely voltage shifted. Also, the I_{CaL} final equation, combining the various gating variables and a static Ca^{2+} concentration-independent Nernst potential in the original Kurata model is substituted for the I_{CaL} formulation in the Ten-Tusscher model [4]. This way, Ca^{2+} handling and the I_{CaL} are appropriately coupled to prevent any sustained accumulation of the cytosolic Ca^{2+} concentration.

$$p_{I_{CaL}}: \{\boldsymbol{p_{I_{CaL}}}[\mathbf{0}], \boldsymbol{p_{I_{CaL}}}[\mathbf{1}], \boldsymbol{p_{I_{CaL}}}[\mathbf{2}], \cdots, \boldsymbol{p_{I_{CaL}}}[\mathbf{5}]\}$$

$$d_{L\infty} = \frac{1}{1 + exp\left(-\frac{V_m + \boldsymbol{p}_{\boldsymbol{I_{CaL}}}[\mathbf{1}]}{\boldsymbol{p}_{\boldsymbol{I_{CaL}}}[\mathbf{2}]}\right)} \tag{39}$$

$$f_{L\infty} = \frac{1}{1 + exp\left(\frac{V_m + \boldsymbol{p}_{\boldsymbol{I_{CaL}}}[\mathbf{3}]}{\boldsymbol{p}_{\boldsymbol{I_{CaL}}}[\mathbf{4}]}\right)} \tag{40}$$

$$f_{Ca\infty} = \frac{K_{mfCa}}{K_{mfCa} + [Ca^{2+}]_{sub}} \tag{41}$$

$$\alpha_{d_L} = -0.02839 \cdot \left[\frac{(V_m + 35)}{exp\left(-\frac{(V_m + 35)}{2.5}\right) - 1}\right] - 0.0849 \cdot \left[\frac{V_m}{exp\left(\frac{-V_m}{4.8}\right) - 1}\right] \tag{42}$$

$$\beta_{d_L} = 0.01143 \cdot \frac{(V_m - 5)}{exp\left(\frac{(V_m - 5)}{2.5}\right) - 1} \tag{43}$$

$$\tau_{d_L} = \frac{1}{\alpha_{d_L} + \beta_{d_L}} \tag{44}$$

$$\tau_{f_L} = 257.1 \cdot exp\left[-\left(\frac{(V_m + 32.5)}{13.9}\right)^2\right] + 44.3$$

$$\tau_{fCa} = \frac{f_{Ca\infty}}{\alpha_{fCa}}$$

$$I_{CaL} = \boldsymbol{p}_{\boldsymbol{I_{CaL}}}[\mathbf{0}] \cdot d_L \cdot f_L \cdot f_{Ca} \cdot 4 \cdot \frac{(V_m - \boldsymbol{p}_{\boldsymbol{I_{CaL}}}[\mathbf{5}])\, F^2}{RT} \cdot \frac{Ca_i \cdot exp\left(\frac{2(V_m - \boldsymbol{p}_{\boldsymbol{I_{CaL}}}[\mathbf{5}])F}{RT}\right) - 0.341 Ca_o}{exp\left(\frac{2(V_m - \boldsymbol{p}_{\boldsymbol{I_{CaL}}}[\mathbf{5}])F}{RT}\right) - 1} \tag{45}$$

Table S5. Initial and fitted parameter values I_{CaL} formulation.

**Parameter**	$\boldsymbol{p}_{\boldsymbol{I_{CaL}}}[\mathbf{0}]$	$\boldsymbol{p}_{\boldsymbol{I_{CaL}}}[\mathbf{1}]$	$\boldsymbol{p}_{\boldsymbol{I_{CaL}}}[\mathbf{2}]$	$\boldsymbol{p}_{\boldsymbol{I_{CaL}}}[\mathbf{3}]$	$\boldsymbol{p}_{\boldsymbol{I_{CaL}}}[\mathbf{4}]$	$\boldsymbol{p}_{\boldsymbol{I_{CaL}}}[\mathbf{5}]$
Original	0.058 [2]	14.1	6	30	5	0
Fitted (Rep.)	0.18723	1.00592	6.38249	29.16061	3.60776	0.09458
% Change	222.81	-92.87	6.37	-2.80	-27.84	

The ionic activity coefficients of 1 (intracellular) and 0.341 (extracellular) in I_{CaL} formulation were recently shown to be problematic by Tomek et al. [5]. Therefore, we implemented an alternative formulation of I_{CaL} with an ionic activity coefficient of 0.63 along with the same fitted parameters and noticed that the resultant current density was merely scaled. However, no voltage shift in the

[2] The value of $\boldsymbol{p}_{\boldsymbol{I_{CaL}}}[\mathbf{0}]$ in the Kurata model of 0.58 was adjusted to qualitatively match the model and experimental peak values prior to fitting. The motivation for this was to accelerate the parameter search time due to a reduced initial variance between model output and experimental data.

peak current density was observed (see Figure S1 below). This is equivalent to scaling the maximum conductance of I_{CaL}. We, therefore, kept our original formulation with the ionic activity coefficient of 0.341 to obviate recalibration of all parameters.

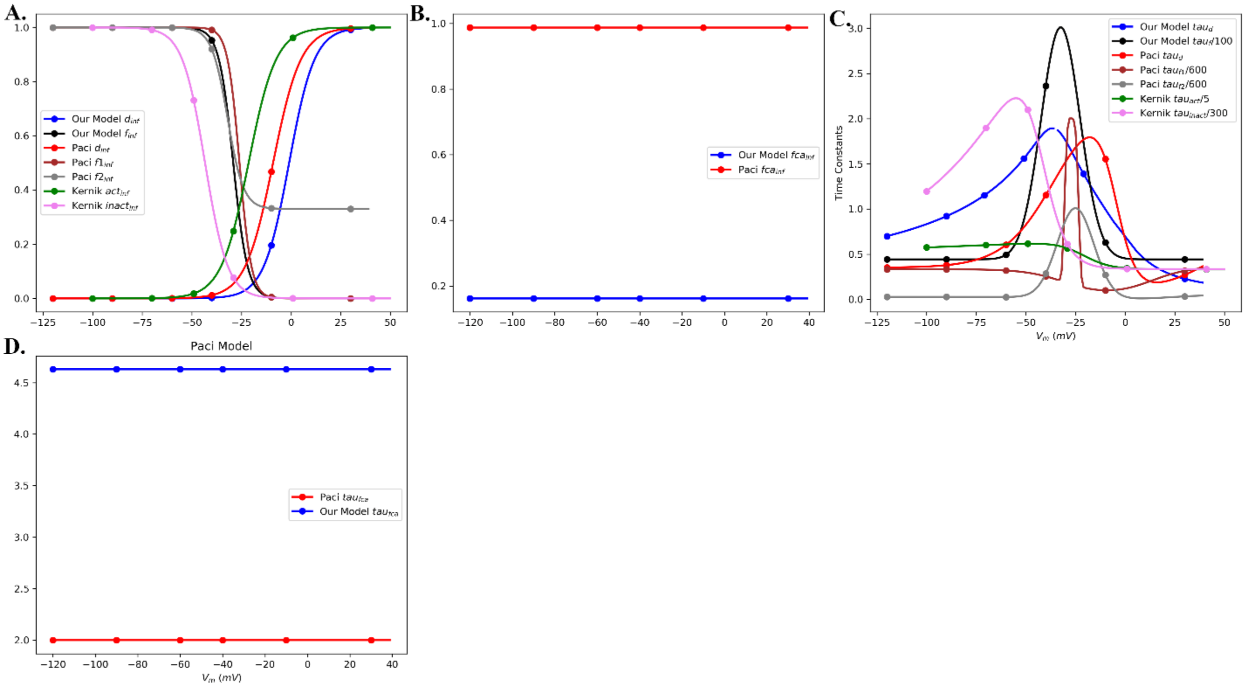

Figure S4. Comparison of I_{CaL} activation, inactivation and corresponding time constants in our model with those of Paci et al. [1] (Paci2018 model) and Kernik et al. [2]

.

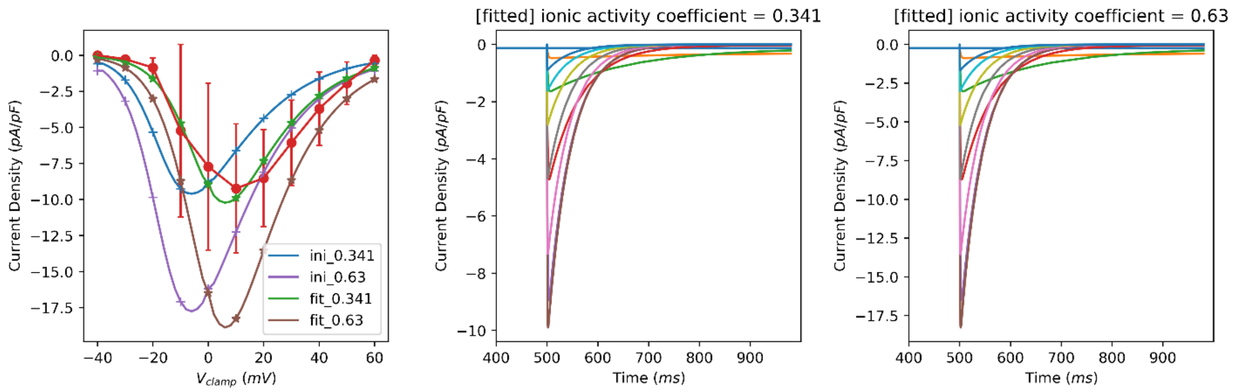

Figure S5. Exploring an ionic activity coefficient (IAC) of 0.63 juxtaposed with an IAC of 0.341 in I_{CaL} formulation.

5. I_f Parametrization (7 free parameters)

$$p_{I_f}: \{\boldsymbol{p_{I_f}}[\mathbf{0}], \boldsymbol{p_{I_f}}[\mathbf{1}], \boldsymbol{p_{I_f}}[\mathbf{2}], \cdots, \boldsymbol{p_{I_f}}[\mathbf{6}]\}$$

$$y_\infty = \frac{1}{1 + exp\left(\frac{V_m + 80.6}{6.8}\right)} \tag{46}$$

$$a_y = exp\left(-\left(\boldsymbol{p_{I_f}}[\mathbf{2}] + \mathbf{p_{I_f}}[\mathbf{3}] \cdot V_m\right)\right) \tag{47}$$

$$b_y = exp\left(\boldsymbol{p_{I_f}}[\mathbf{4}] + \boldsymbol{p_{I_f}}[\mathbf{5}] \cdot V_m\right) \tag{48}$$

$$\tau_y = \frac{\boldsymbol{p_{I_f}}[\mathbf{6}]}{a_y + b_y} \tag{49}$$

$$I_{fK} = \boldsymbol{p_{I_f}}[\mathbf{1}] \cdot y \cdot (V_m - E_K) \tag{50}$$

$$I_{fNa} = \boldsymbol{p_{I_f}}[\mathbf{0}] \cdot y \cdot (V_m - E_{Na}) \tag{51}$$

$$I_f = I_{fK} + I_{fNa} \tag{52}$$

Table S6. Initial and fitted parameter values I_f formulation.

**Parameter**	$\boldsymbol{p_{I_f}}[\mathbf{0}]$	$\boldsymbol{p_{I_f}}[\mathbf{1}]$	$\boldsymbol{p_{I_f}}[\mathbf{2}]$	$\boldsymbol{p_{I_f}}[\mathbf{3}]$	$\boldsymbol{p_{I_f}}[\mathbf{4}]$	$\boldsymbol{p_{I_f}}[\mathbf{5}]$	$\boldsymbol{p_{I_f}}[\mathbf{6}]$
Original	0.02343	0.01456	2.9	0.04	3.6	0.11	4000
Fitted (Rep.)	0.050297	0.07028	1.68827	0.036728	5.176284	0.053505	4010.313
% Change	114.67	382.69	-41.78	-8.18	43.79	-51.36	0.26

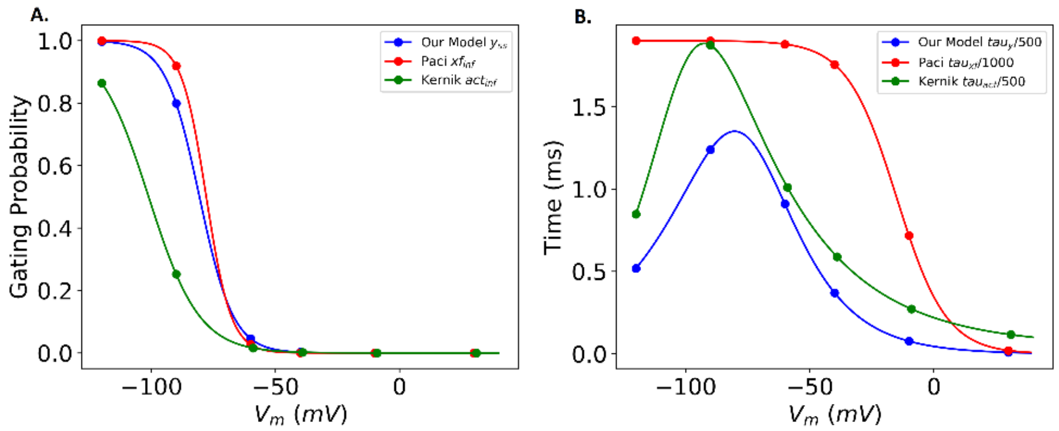

Figure S6. Comparison of I_f inactivation and corresponding time constants in our model with those of Paci et al. [1] (Paci2018 model) and Kernik et al. [2]

6. Other Model Related Parameters

Table S7. Maximum conductance values of the rest of the ion currents.

Ionic Current	Parameter	Our Model Value	Original Model Value	% Scaling[1]
Ultrarapid delayed rectifier potassium current, I_{Kur}	K_I_{Kur}	0.8	1	80
Sodium-calcium exchanger current, I_{NaCa} (pA/pF)	I_{NaCa_max}	1620	1600	101.25
Sodium-potassium pump current, I_{NaK} (pA/pF)	I_{NaK_max}	13.73	6.0	228
Background sodium current, I_{bNa} (nS/pF)	G_b_{Na}	0.001078	0.000674	159.94
Background calcium current, I_{bCa} (nS/pF)	G_b_{Ca}	0.001808	0.00113	160
Slow delayed rectifier potassium current, I_{Ks} (nS/pF)	G_ks	0.0387	0.129	30
Sarcolemmal calcium pump current, I_{pCa} (pA/pF)	I_{pCa_max}	1.43	0.275	520
Inward rectifier potassium current, I_{K1}	K_I_{K1}	0.18	1	18
Acetylcholine-activated potassium current, I_{KACh}	K_I_{Kach}	1.0	1.0	100
Fast inward sodium current, I_{Na}^{*} (mS/μF)	G_{Na}	10.18724	12	84.89
Slow delayed rectifier potassium current, I_{Kr}^{*}	K_I_{Kr}	0.025	1	2.5
L-type calcium current, I_{CaL}^{*} (nS/pF)	$G_{Ca,L}$	0.20595128	0.58	35.5

Transient outward potassium current, I_{to}^{*} (mS/μF)	$G_{to,fast}$<br>$G_{to,slow}$	0.000687<br>0.099254	0.0014<br>0.0376	49.09<br>264
Hyperpolarization activated pacemaker current, I_f^{*} (nS/pF)	$G_{f,Na}$<br>$G_{f,K}$	0.04275<br>0.0597	0.0145654<br>0.0234346	293<br>255

* GA fitted currents scaling adjustment limited to ±25%

[1]The % parameter scaling may appear larger because of differences in cell volume and cell capacitance of our model with respect to the original cell parameters.

Table S8. Cell-related constants used in the model and original references.

Model Constant	Formulation Source	Description/ Value
Cell Volume, V_{cell}	Same as in Courtemanche *et al.* [6]	Human cell geometry, 20100μm^3
Intracellular subspace volume, V_{sub}	Same as in Kurata *et al.* [7]	Compartment proportions maintained since calcium dynamics adopted from Kurata *et al.* [7], a rabbit cell model. The cell dimensions are rather adopted from Courtemanche *et al.* [6], a human cell model.
Intracellular volume, V_i		
NSR volume, V_{up}		
JSR volume, V_{rel}		
Maximum Calcium pump current, I_{pCa_max}		
Calcium Buffering constants		
Maximum SR uptake rate		0.0075mM/ms
Maximum SR release rate		50 ms^{-1}
SR transfer flux time constant		27.5ms
Maximum sodium-calcium exchanger conductance, I_{NaCa_max}		100pA/pF
Maximum sodium-potassium exchanger conductance, I_{NaK_max}		4.3pA/pF
Membrane Capacitance, C_m		100pF

Table S9. Steady state (SS) initial conditions for model variables

Model Variable	SS initial condition
V_m	-66.643468720486
m	0.049124825877
h	0.727700325891
j	0.894208866121
$[Na^+]_i$	9.680699292047
$[K^+]_i$	91.709001163473
x_{tof}	0.005930308293
y_{tof}	0.981076047295
x_{tos}	0.005925315068
y_{tos}	0.257724187021
p_{aF}	0.005855419715
p_{aS}	0.036098779153
p_i	0.868561127740
y_f	0.065137562713
d	0.000033876121
f	0.997882750596
f_{Ca}	0.547948046949
f_{tc}	0.043582776022
f_{tmc}	0.449383699221
f_{tmm}	0.486436847737
f_{cmi}	0.087712453951
f_{cms}	0.108049141579
f_{cq}	0.028782461964
$[Ca^{2+}]_i$	0.000228812553
$[Ca^{2+}]_{sub}$	0.000288302762
$[Ca^{2+}]_{up}$	0.825695731160
$[Ca^{2+}]_{rel}$	0.024701468338
x_s	0.048136026971
u_a	0.021931284659
u_i	0.969241564523

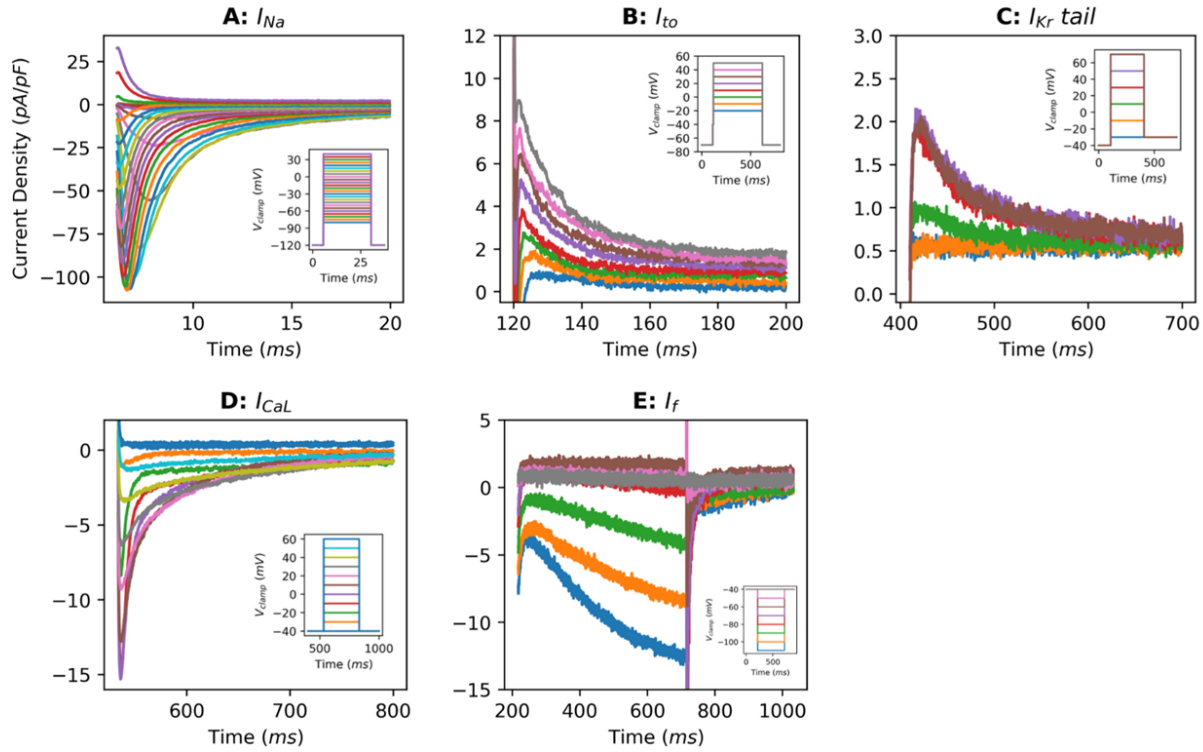

Figure S7. Representative experimental current traces recorded during patch clamping.

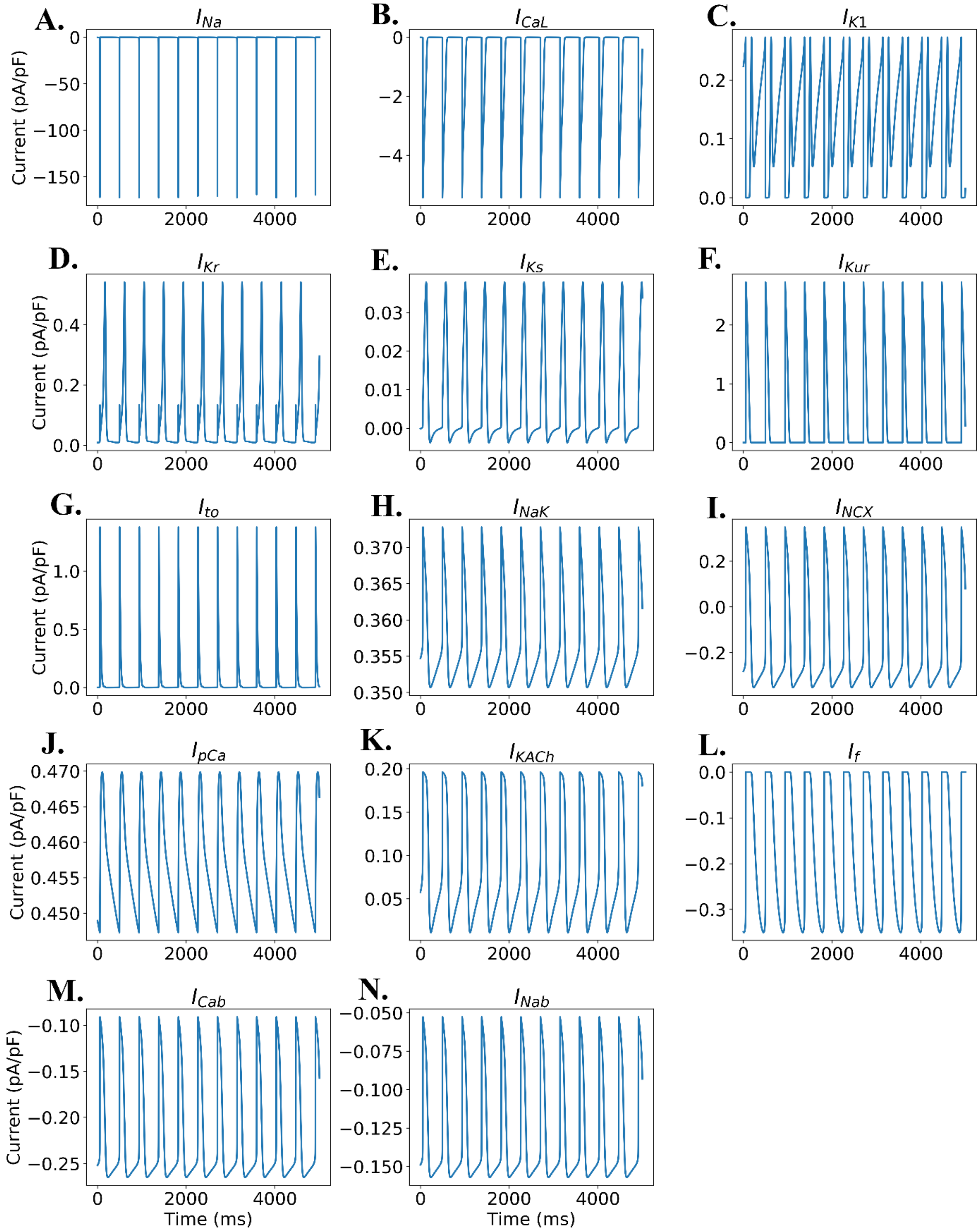

Figure S8. Various ionic currents during spontaneous AP activity in the model.

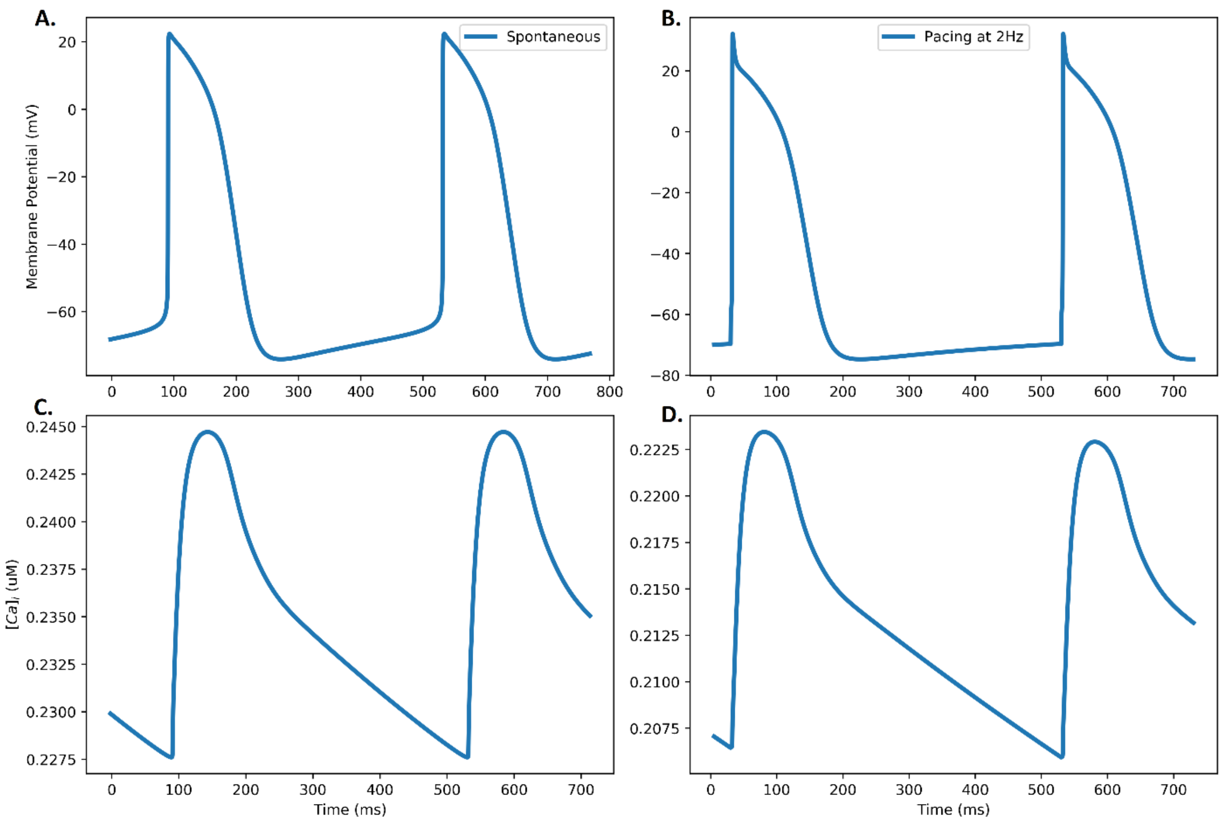

Figure S9. A) Spontaneous AP morphology and C) corresponding intracellular calcium transients; B) AP elicited by 2 Hz pacing stimulus and D) corresponding intracellular calcium transients.

Table S10. Intracellular calcium transient parameters in our model during spontaneous AP.

**Parameter**	**Our Model**	**Kernik et al. [2]**	**Paci et al. [1] (Paci2018 model)**
Rise time from 10% to 50% of peak value (RT_{1050}) (ms)	7.45	22.6	47.7
Rise time from 10% to 90% of peak value (RT_{1090}) (ms)	30.7	94.6	106
Decay time from 90% to 10% of peak value (DT_{9010}) (ms)	127.25	297.3	367.6
Freq of spontaneous activation (Hz)	2.26	1.02	0.65
Time to peak (T_{peak}) (ms)	65.6	202.4	165.9
